# Flexible Organic Photovoltaic‐Powered Hydrogel Bioelectronic Dressing With Biomimetic Electrical Stimulation for Healing Infected Diabetic Wounds

**DOI:** 10.1002/advs.202307746

**Published:** 2023-12-25

**Authors:** Yi‐Wei Hu, Yu‐Heng Wang, Fang Yang, Ding‐Xin Liu, Guang‐Hao Lu, Sheng‐Tao Li, Zhi‐Xiang Wei, Xiang Shen, Zhuang‐De Jiang, Yi‐Fan Zhao, Qian Pang, Bai‐Yang Song, Ze‐Wen Shi, Shareen Shafique, Kun Zhou, Xiao‐Lian Chen, Wen‐Ming Su, Jia‐Wen Jian, Ke‐Qi Tang, Tie‐Long Liu, Ya‐Bin Zhu

**Affiliations:** ^1^ Health Science Center Ningbo University Ningbo 315211 P. R. China; ^2^ Orthopaedic Oncology Center of Changzheng Hospital Naval Medical University Shanghai 200003 P. R. China; ^3^ Faculty of Electrical Engineering and Computer Science Ningbo University Ningbo 315211 P. R. China; ^4^ State Key Laboratory of Electrical Insulation and Power Equipment Xi'an Jiaotong University Xi'an 710049 P. R. China; ^5^ CAS Key Laboratory of Nanosystem and Hierarchical Fabrication National Center for Nanoscience and Technology University of Chinese Academy of Sciences Beijing 100049 P. R. China; ^6^ The Research Institute of Advanced Technologies Ningbo University Ningbo 315211 P. R. China; ^7^ State Key Laboratory for Manufacturing Systems Engineering Xi'an Jiaotong University Xi'an 710049 P. R. China; ^8^ School of Physical Science and Technology Ningbo University Ningbo 315211 P. R. China; ^9^ Shenzhen Institute of Aggregate Science and Technology The Chinese University of Hong Kong Shenzhen Shenzhen 518172 P. R. China; ^10^ Printable Electronics Research Center & Nano‐Device and Materials Division Suzhou Institute of Nano‐Tech and Nano‐Bionics Nano Chinese Academy of Sciences Suzhou 215123 P. R. China; ^11^ Institute of Mass Spectrometry School of Material Science and Chemical Engineering Ningbo University Ningbo 315211 P. R. China

**Keywords:** conducting hydrogel, electrical stimulation, flexible photovoltaic cells, proteomics, wound dressing

## Abstract

Electrical stimulation (ES) is proposed as a therapeutic solution for managing chronic wounds. However, its widespread clinical adoption is limited by the requirement of additional extracorporeal devices to power ES‐based wound dressings. In this study, a novel sandwich‐structured photovoltaic microcurrent hydrogel dressing (PMH dressing) is designed for treating diabetic wounds. This innovative dressing comprises flexible organic photovoltaic (OPV) cells, a flexible micro–electro–mechanical systems (MEMS) electrode, and a multifunctional hydrogel serving as an electrode–tissue interface. The PMH dressing is engineered to administer ES, mimicking the physiological injury current occurring naturally in wounds when exposed to light; thus, facilitating wound healing. In vitro experiments are performed to validate the PMH dressing's exceptional biocompatibility and robust antibacterial properties. In vivo experiments and proteomic analysis reveal that the proposed PMH dressing significantly accelerates the healing of infected diabetic wounds by enhancing extracellular matrix regeneration, eliminating bacteria, regulating inflammatory responses, and modulating vascular functions. Therefore, the PMH dressing is a potent, versatile, and effective solution for diabetic wound care, paving the way for advancements in wireless ES wound dressings.

## Graphical abstract

A sandwich‐structured photovoltaic microcurrent hydrogel dressing (PMH dressing) for chronic bacteria‐infected diabetic wounds is developed. PMH dressing generates a biomimetic current under indoor light, which is delivered to the wound through the hydrogel interface. PMH dressing significantly enhances the healing of infected diabetic wounds through improved extracellular matrix regeneration, bacterial clearance, and regulation of inflammatory responses and vascular functions.

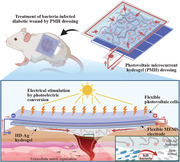

## 1 Introduction

Diabetic wounds represent a profoundly debilitating complication, afflicting ≈25% of individuals with type II diabetes during their lifetimes.^[^ ^1^ ^]^ Unfortunately, statistics suggest that a lower limb is amputated every 30 s worldwide due to non‐healing diabetic wounds.^[^ ^2^ ^]^ Despite the availability of various treatment alternatives, such as antibiotics, nitric oxide therapy, biomaterial gels, and nanozymes, effective treatment options are limited.^[^ ^3^ ^]^ Typically, diabetic wounds are caused by severe bacterial infection, abnormal inflammation, impaired collagen production, and reduced epidermal development.^[^ ^3a^ ^]^ Intriguingly, diabetic wounds also exhibit a weakening of the endogenous electric field (EF).^[^ ^4^ ^]^ The endogenous EF plays a critical role in wound healing by guiding the migration of electroactive cells such as fibroblasts and epithelial cells. Upon wound occurrence, a microcurrent, referred to as the “injury current,” flows from the wound's edge (the anode) toward the center (the cathode).^[^ ^5^ ^]^ Numerous studies have demonstrated that electrical stimulation (ES) promotes wound healing in diabetic patients with chronic non‐healing wounds.^[^ ^4^, ^6^ ^]^ A double‐blind randomized control trial that encompassed administering home‐based ES for diabetic foot ulcers revealed a significant 22% reduction in wound area in the intervention group after 4 weeks.^[^ ^7^ ^]^ Commercialized ES dressings such as PosiFect and Procellera, based on metallic electrodes, have been developed to address non‐healing wounds.^[^ ^8^ ^]^ Recently, wound dressings capable of delivering a directional electric field to treat acute wounds have been reported.^[^ ^9^ ^]^ However, achieving a consistent ES analogous to the injury current is challenging because of the absence of wearable power supplies and circuit guidance in bioelectric dressings.^[^ ^10^ ^]^ The human body, consisting of soft, water‐rich tissue, stands in stark contrast to lab‐made bioelectronic devices constructed using rigid, dry electronic components. This disparity has hindered the desired seamless interfacing between biological tissue and electrodes.^[^ ^11^ ^]^ Therefore, optimizing the tissue–electrode interface is crucial for treating diabetic wounds of varying sizes, shapes, and depths. Therefore, forming a compatible and stable interface can facilitate electrical communication between neural tissues and external electronics, notably enhancing the efficiency of ES therapy.^[^ ^12^ ^]^

Wearable and implantable electronics have attracted considerable attention in the health industry owing to their distinct advantages, such as facilitating seamless connections between humans and information, applicability to personal healthcare monitoring systems, and compatibility with smart textiles.^[^ ^13^ ^]^ The field of wearable bioelectronics is particularly interested in advancing flexible and stretchable sensors and functional circuits on 3D freeform surfaces.^[^ ^14^ ^]^ These components are designed to capture and interpret various bio‐signals from our bodies for medical applications. Recently, a tactile sensor was developed using a micro‐pyramid‐patterned double‐network ionic hydrogel, capable of measuring variations in a triboelectric output signal.^[^ ^15^ ^]^ In addition, ultrathin AgNWs/parylene hybrid films with an adjustable 3D structure shaped through laser cutting have been applied for monitoring electrocardiogram signals and sweat levels.^[^ ^16^ ^]^ Various techniques, such as photopatterning, adaptive 3D printing, and transfer printing, have been employed to develop functional circuits. A high‐density transistor array (up to 347 transistors cm^−2^) was developed using dielectric and semiconductor materials through the photopatterning process, contributing to the development of stretchable digital circuits for integration into electronic skin.^[^ ^14b^ ^]^ Despite its potential, photopatterning is limited because of high hardware requirements and technical complexities. By contrast, adaptive 3D printing is a closed‐loop technique that integrates real‐time feedback control with the direct ink writing of functional materials to incorporate devices on moving freeform surfaces.^[^ ^17^ ^]^ Another efficient method for fabricating 3D electronic devices is transfer printing, which employs stamps to lift devices from donor substrates and imprint them onto receiver substrates.^[^ ^18^ ^]^ This is a cost‐effective technique for generating complex geometries with high precision and at a low cost.

While conventional wearable medical devices rely on chemical batteries (button batteries) and commercial electricity for power, the requirement of frequent replacement and maintenance renders them cumbersome and cost‐ineffective. Self‐powered devices, such as enzymatic biofuel cells (EBFCs), radio frequency energy harvesting (RFEH) methods, and organic photovoltaic (OPV) cells, have emerged as potential solutions for gathering operational power from renewable energy sources, offering promising avenues for wireless self‐powered health diagnostics and therapeutics.^[^ ^19^ ^]^ EBFCs are employed to harvest energy in biofluid models and function as self‐powered electrochemical glucose sensors.^[^ ^20^ ^]^ RFEH methods encompass using rectennas, which combine an antenna and a rectifier, to convert radio frequency (RF) energy into useful direct current power, eliminating the need for batteries or wires.^[^ ^21^ ^]^ However, the efficiency of these devices is affected by the stability of enzymes or interference from other RF sources, limiting their suitability for high‐power applications. OPV cells, ideal for indoor energy harvesting,^[^ ^22^ ^]^ have gained attention as potential power solutions capable of generating sufficient electrical charge for wearable medical devices. For example, OPV cells have been utilized for the ES of peripheral nerves or retinal cells, with the aim of restoring neural functions.^[^ ^23^ ^]^ In the context of bioelectrical wound dressings, OPV cells are superior to other wearable power systems owing to their distinctive advantages. They efficiently convert light energy into electrical energy, providing a self‐sustaining power source—an essential criterion in the design of bioelectrical wound dressings. This functionality aligns with the broader development trend of wearable devices capable of generating self‐sustaining, biologically‐responsive electrical stimulation from natural light energy. Unlike piezoelectric and triboelectric nanogenerators, OPV cells are wireless and self‐powered, eliminating the need for external power supplies and resulting in a more compact and convenient wearable system.^[^ ^24^ ^]^ Owing to their durability and stability under diverse environmental conditions, OPV cells ensure consistent performance even under bodily conditions.^[^ ^25^ ^]^ Further, the biocompatibility of OPV cells ensures their safety for usage in direct contact with biological tissues.^[^ ^26^ ^]^ Lastly, non‐fullerene materials endow OPV cells with advantages such as a broader absorption spectrum, adjustable energy levels, and enhanced resistance to degradation.^[^ ^27^ ^]^ Therefore, OPV cells represent a reliable, efficient, cost‐effective, and safe power source for bioelectrical wound dressings.

Hydrogels have been extensively investigated in the field of tissue engineering and wound dressing owing to their exceptional biocompatibility.^[^ ^28^ ^]^ The soft and flexible nature of hydrogels minimizes mechanical mismatches with biological tissues while maintaining a wet environment with an abundance of ions that mimic physiological conditions. Moreover, hydrogels exhibit remarkable versatility in electrical, mechanical, antimicrobial, and biological properties, making them particularly suitable for serving as an interface between wound tissue and electronics.^[^ ^29^ ^]^ Hydrogels, such as hyaluronan/chitosan, possess immunomodulatory properties.^[^ ^30^ ^]^ These natural polymers can facilitate the transition of macrophages from an inflammatory to a proliferative (M2) phenotype, thereby accelerating diabetic wound healing. In recent years, commercially available wound dressings contain silver nanoparticles (AgNPs) that enhance their bactericidal activity.^[^ ^31^ ^]^ AgNPs have demonstrated efficacy against a variety of microorganisms, including multi‐resistant bacteria, exhibiting great potential in the treatment of diabetic wounds.^[^ ^32^ ^]^

**Figure 1:**
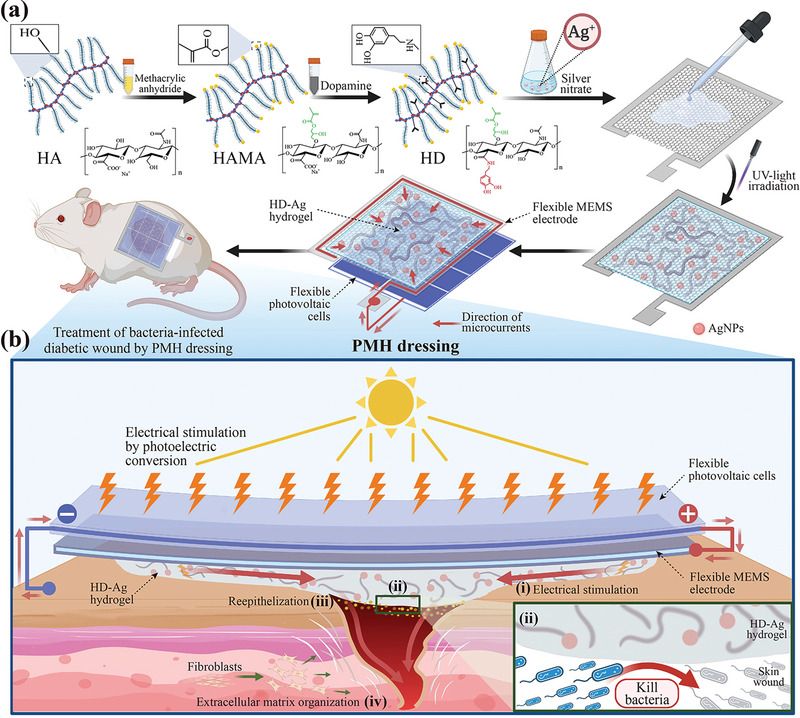
a) The fabrication process of the PMH dressing involves synthesizing dopamine‐modified hyaluronic acid methacryloyl (HAMA), followed by the preparation of conductive HD‐Ag hydrogel onto a flexible MEMS electrode. The PMH dressing is obtained by connecting flexible OPV cells to the MEMS electrode, designed to provide biomimetic electrical stimulation under light conditions. The PMH dressing is then applied to a bacteria‐infected skin wound on diabetic mice. b) The illustration below outlines the ways the PMH dressing can accelerate wound healing processes among diabetics: b‐i) providing biomimetic electrical stimulation, b‐ii) inhibiting bacteria proliferation, b‐iii) accelerating reepithelization, and b‐iv) enhancing extracellular matrix organization and tissue remodeling.

This paper proposes a novel protocol for developing a photovoltaic wound dressing, referred to as the photovoltaic microcurrent hydrogel (PMH) dressing, and identifying key protein alterations involved in diabetic wound healing. The PMH dressing is designed to satisfy the following criteria: a) the ability to provide biomimetic ES; b) exhibiting antibacterial properties; c) capable of establishing seamless contact with irregular wound beds; and d) non‐invasive, wearable, and user‐friendly. The PMH dressing comprises flexible OPV cells as a portable battery, a flexible micro–electro–mechanical systems (MEMS) electrode as a guidance circuit,^[^ ^33^ ^]^ and a conductive hydrogel as an electrode–wound interface (**Figure** **1**). First, the flexible OPV cells, consisting of Ag‐grid MEMS substrates and non‐fullerene organic solar cells, are used to support ES under light conditions.^[^ ^25^, ^34^ ^]^ Second, the MEMS electrode, fabricated using transfer printing techniques, can guide micro‐currents analogous to injury currents. Third, the conductive hydrogel is employed as an interface between the MEMS electrode and the wound site. In vivo experiments are conducted in diabetic mice with bacteria‐infected wounds to evaluate the efficacy of the PMH dressing in accelerating wound healing. Finally, we discern the possible molecular mechanisms through which the PMH dressing facilitates the healing of chronic wounds.

## 2 Results and Discussion

### 2.1 Design, Fabrication, and Characterization of the PMH Dressing

**Figure 2:**
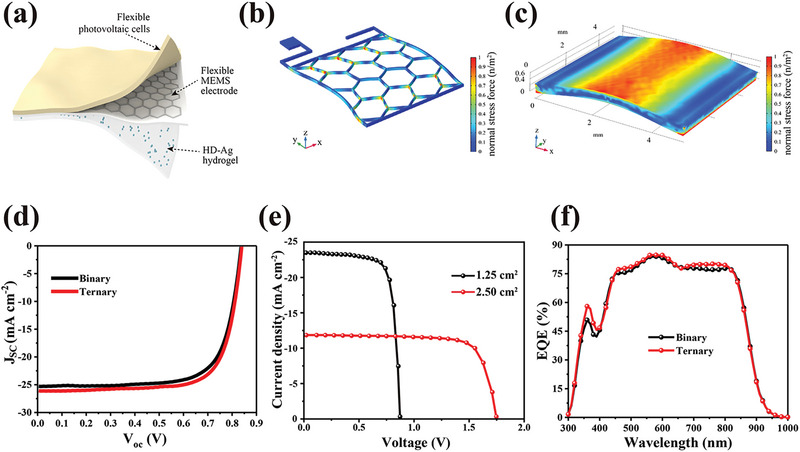
a) The schematic of the sandwich‐structured PMH dressing, composed of large‐area flexible OPV cells on the top, a flexible MEMS electrode in the middle, and a hydrogel in contact with skin at the bottom. b) Stress finite element analysis of the MEMS electrode. c) Finite element simulation of mechanical properties for organic semiconductor materials. d) *J–V* curves of binary and ternary OPV cells. e) The *J–V* features of the OPV cells with different areas. f) The external quantum efficiency (EQE) spectra of the OPV cells.

The wearable PMH dressing employs a sandwich‐type architecture, featuring flexible OPV cells at the top and a flexible MEMS electrode in the middle (**Figure** **2**a). At its base, serving as the wound interface, is a multifunctional composite hydrogel composed of dopamine‐modified HAMA (HAMA‐DA) and in situ‐formed AgNPs (HD‐Ag hydrogel, Figure 1a). As illustrated in Figure 1b, under light conditions, OPV cells convert light energy into electric power. The MEMS electrode is connected to the OPV cells, and specially designed microcircuits on the MEMS electrode guide the direction of the electric current, achieving a biomimetic injury current that flows from the wound's edge toward the central region. The HD‐Ag hydrogel in the PMH dressing maintains conformal contact over time, reducing interfacial impedance and delivering microcurrent stimulation from the MEMS electrode to the wound area.

PM6 and Y6 constitute the active layers of the devices, responsible for converting light into electricity. Since their introduction in 2019, PM6 and Y6 materials have become iconic in the field of organic optoelectronics.^[^ ^35^ ^]^ Their photovoltaic conversion efficiency significantly surpasses that of the fullerene system, demonstrating excellent stability and a broad spectrum of light absorption.^[^ ^36^ ^]^ In this study, we introduced thermoplastic elastomer (TPE) to enhance mechanical properties, such as stable interfacial adhesion and bending durability, further improving optoelectronic properties (Figure S1a–c, Supporting Information). Small‐area devices were fabricated using patterned ITO glass; while, large‐area flexible organic solar cells were printed on a flexible electrode using a sequential slot die method. Finite element simulation revealed the superior elasticity of the flexible MEMS electrode and the organic semiconductor light‐absorbing layer (Figure S2b–d, Supporting Information). This processing method, employed in previous reports,^[^ ^22^, ^37^ ^]^ has yielded numerous high‐performance optoelectronic devices. Stress is evenly distributed on the edge of the silver grid, ensuring apparatus durability and resilience (Figure 2b). Under pressure, the organic semiconductor layer's stress gradually disperses, demonstrating strong tensile and compression resistance and making it suitable as a dressing on moving skin tissues (Figure 2c). As depicted in Figure 2d, the open‐circuit voltage and short‐circuit current of the ternary materials are delineated. The open‐circuit voltage remains stable at ≈1.0 V, while the short‐circuit current is maintained at the standard of 20 mA cm^−2^, a level consistent with our earlier studies that confirm it does not lead to significant skin burns.^[^ ^25^ ^]^ The JV curves of our single‐device and series‐device modules are also presented (Figure 2e). The ternary system ensures a stable supply of voltage required for ES.^[^ ^38^ ^]^ As shown in Figure 2f, the EQE spectra for the verified devices exhibit power conversion efficiency (PCE) test accuracy. The broad absorption peak beyond 900 nm results from the Y6 molecule. The overlapping voltage in larger devices suggests potential use over larger skin areas.

For wounds with a diameter of ≈10 mm, an electrical field of 1.0 V (equivalent to 100 mV mm^−1^) is reported as suitable for eliciting a biological response.^[^ ^9^, ^39^ ^]^ Under typical indoor light conditions (2700 K LED array with a luminous intensity of 1000 lux), OPV cells can generate a stable open‐circuit voltage of 1.07–1.12 V.^[^ ^22b^ ^]^ The 0.04 cm^2^ small‐area flexible device achieves an impressive PCE of up to 16%; while, the 1.25 cm^2^ large‐area flexible device attains a PCE of up to 13%, demonstrating the advanced level of device technology.^[^ ^40^ ^]^ Due to the excellent flexibility of poly ethylene terephthalate (PET) loaded with silver grids (Young's modulus of 0.45 MPa), the silver grid composite substrate devices (PET‐solar cells and PET‐electrode) display a reduced bending pattern and adapt well to the skin surface. Moreover, given that biological safety is paramount for any wound dressing intended for medical use, our research assesses the potential toxicity of the bioelectronic components integrated into the novel dressing. Our findings confirm the excellent biocompatibility of the OPV cells and MEMS electrodes (Figure S3, Supporting Information), consistent with our previous study.^[^ ^25^ ^]^

An ES therapy for wound healing, capable of providing a biomimetic EF or injury current from the periphery to the center of the wound, is often considered the optimal solution. The EF intensity at skin injury sites typically falls within the range of 100–200 mV mm^−1^.^[^ ^9^, ^41^ ^]^ For instance, the EF in mouse skin wounds is measured at 122 ± 9 mV mm^−1^.^[^ ^42^ ^]^ Thus, our aim is to achieve an ES intensity of ≈100 mV mm^−1^. Most current portable ES wound dressings utilize a stimulation intensity equivalent to the physiological EF, and some innovative products use hollow ring electrodes to generate a biomimetic EF (refer to Table S2, Supporting Information). However, due to the variable depth of wound tissues, it is challenging for regular‐shaped metal or silicone rubber electrodes to produce uniform ES, potentially leading to localized aggregation of electrolysis products and subsequent chemical burns.

The development of miniaturized and wireless power supply devices is crucial to the advancement of ES wound dressings. Traditional power supply devices for electric stimulation are often characterized by bulkiness, tethering, and inconvenience for patients, thereby limiting their applicability and adoption.^[^ ^43^ ^]^ The advent of compact, wireless power supply devices has paved the way for the development of user‐friendly, efficient, and portable ES therapies for wound healing.^[^ ^44^ ^]^ Details on the performance and applications of various energy‐harvesting strategies currently available for wearable biomedical devices are summarized in Table S3, Supporting Information. OPV cells fabricated with non‐fullerene acceptor (NFA) materials offer unique benefits for ES wound dressings. NFA materials are marked by their potential for structural customization, high property tunability, intense absorption of visible and near‐infrared light, and remarkable n‐type semiconducting characteristics.^[^ ^45^ ^]^ Further, these OPV cells offer a range of benefits including cost‐effectiveness, flexibility, semi‐transparency, high mass power density, short energy payback time, and compatibility with large‐area printing processes, making them ideal for scalable production.^[^ ^46^ ^]^ With the surge of biomedical and health industry, a variety of organic semiconductors with excellent biocompatibility and reliable diagnostic/therapeutic functions is developed.^[^ ^47^ ^]^ The OPV cells applied in this study are flexible, biologically safe, and capable of providing sufficient voltage for the PMH dressing, rendering them highly suitable and promising self‐powering devices for innovative wearable bioelectronics. In the operating mode of the PMH dressing, OPV cells and MEMS electrodes consistently generate a biomimetic EF, stimulating the wound through microcircuits on the MEMS electrode surface and the conductive hydrogel. This model proves particularly beneficial for chronic wound healing as the bioactive hydrogel interface reduces the mechanical strength mismatch between tissue and electrode, delivering uniform and stable ES to the wound.

### 2.2 Synthesis and Material Properties of HD‐Ag Hydrogel Interface

**Figure 3:**
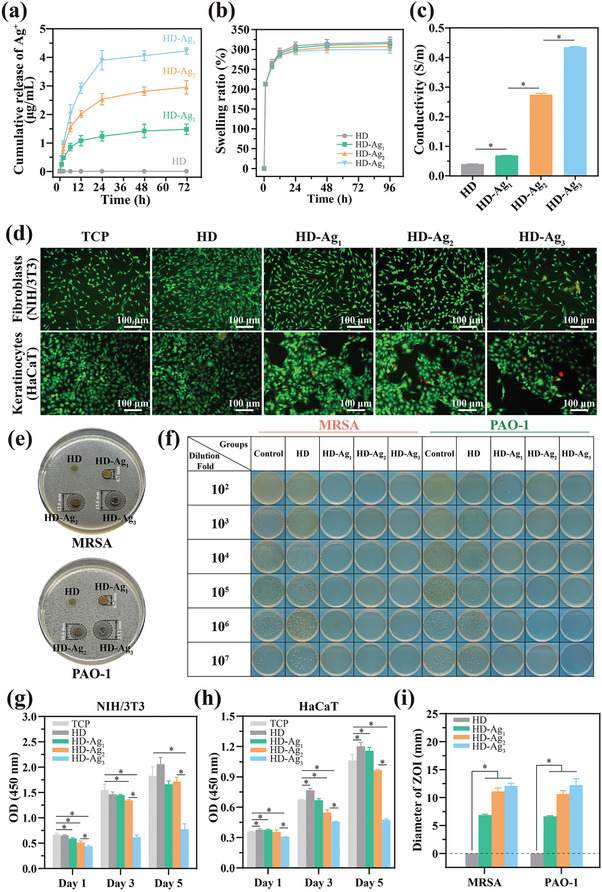
Studies of the physical properties, cytocompatibility, and antibacterial activity of the HD‐Ag hydrogels. a) The release profile of Ag^+^ ions, b) swelling ratio, and c) electrical conductivity of the HD‐Ag hydrogels. d) Representative live/dead stain images of fibroblasts and keratinocytes co‐cultured with the hydrogels for 3 days; green represents live cells, while red represents dead cells. Cells cultured on a tissue culture plate (TCP) were used as control. e,i) Representative photographs and quantification analysis of the inhibition zone against MRSA or PAO bacteria by different hydrogel disks. f) Quantitative analysis of the MRSA and PAO colonies incubated with each hydrogel for 24 h. The initial number for two bacterial types was 5 × 10^7^ in this experiment. g,h) Cell proliferation analysis of fibroblasts and keratinocytes co‐cultured with the HD‐Ag hydrogels for 1, 3 and 5 days. The data were presented as mean ± SD (*n* = 3, *p < 0.05).

Hydrogels possess exceptional biomaterial compatibility, high water content, softness, and adaptable functionality, rendering them ideal bridging materials for achieving seamless connections between MEMS electrodes and wound tissue. In this study, we formulated the HD‐Ag hydrogel, which serves as an interface between the MEMS electrode and wound tissues. It is composed of a combination of HAMA‐DA and Ag nanoparticles. We developed a series of HD‐Ag hydrogels by progressively increasing the concentration of Ag^+^ ions from 1 to 3 mg mL^−1^, which were denoted as HD‐Ag_1_, HD‐Ag_2_, and HD‐Ag_3_, respectively. HD represents the hydrogel without AgNPs. The fabrication process and characterizations of the HD‐Ag hydrogel are detailed in Figures S4–S8, Supporting Information). The HD‐Ag hydrogels were observed to release Ag^+^, with Ag concentrations in the range of 1–5 ppm detected after 12 h (**Figure** **3**a). This aligns with the effective bactericidal concentration of 0.1–5.4 ppm, suggesting that the HD‐Ag series hydrogels may possess effective antibacterial capabilities.^[^ ^48^ ^]^

Ag has long been recognized as an effective treatment for burn wounds, dating back to ancient times and still in use today.^[^ ^49^ ^]^ Recently, Ag has been engineered as a nanomaterial due to its enhanced efficacy in biomedical applications and antimicrobial properties.^[^ ^50^ ^]^ AgNPs undergo slow oxidation reactions to generate silver ions (Ag+), exhibiting antibacterial activity while avoiding adverse reactions from a sudden local release of high concentrations of Ag^+^.^[^ ^51^ ^]^ Through light‐induced reduction reactions, a rapid and uniform reduction of Ag^+^ in situ can be achieved, enabling the even distribution of AgNPs within the hydrogel system, and this uniform distribution of particles ensures good conductivity of the hydrogel.^[^ ^52^ ^]^ In the present study, while photo‐crosslinking occurred in the HD hydrogel, Ag^+^ ions were converted in situ into evenly distributed AgNPs within the hydrogel via a reduction reaction mediated by UV light irradiation and catechol groups.^[^ ^53^ ^]^ This formulation ensured that the HD‐Ag hydrogel maintained reliable conductivity while allowing precise control of Ag^+^ concentrations. In addition, HD‐Ag hydrogels exhibited favorable swelling properties (Figure 3b). Their water absorption capacity enabled effective absorption of effluents, maintaining a moist wound environment that prevented bacterial infection and accelerated the healing process. Further, the excellent electrical conductivity of HD‐Ag hydrogels made them a suitable interface between biological tissues and bioelectronics (Figure 3c).

### 2.3 Biocompatibility and Antibacterial Properties of HD‐Ag Hydrogel Interface

Outstanding biocompatibility is a fundamental property of hydrogels that makes them suitable as interfaces between tissue and bioelectrodes. In this study, we assessed the biocompatibility of HD‐Ag hydrogels using basal layer keratinocytes (HaCaT) and dermal fibroblasts (NIH/3T3) through live/dead staining (Figure 3d; Figure S9, Supporting Information) and the cell counting kit‐8 (CCK‐8) assay (Figure 3g,h). We also conducted subcutaneous embedding experiments (Figure S10, Supporting Information). Our results confirmed the good biocompatibility of HD, HD‐Ag_1_, and HD‐Ag_2_ hydrogels, whereas significant toxicity was associated with the HD‐Ag_3_ hydrogel. Diabetic wounds were characterized by hard‐to‐heal skin ulcers and bacterial infections, commonly caused by Methicillin‐resistant *Staphylococcus aureus* (MRSA, ATCC 43300) and *Pseudomonas aeruginosa* (PAO, ATCC 15962).^[^ ^54^ ^]^ HD‐Ag hydrogel exhibits excellent antibacterial properties, primarily attributed to the presence of AgNPs distributed within the hydrogel (Figure 3f,i). Among these candidates, HD‐Ag_2_ emerged as the most promising due to its impressive conductivity, satisfactory biocompatibility, and antibacterial properties. Therefore, we chose to incorporate HD‐Ag_2_ into the PMH dressing for further research.

### 2.4 In Vivo Effect of the PMH Dressing on Bacteria‐Infected Diabetic Wound Healing

**Figure 4:**
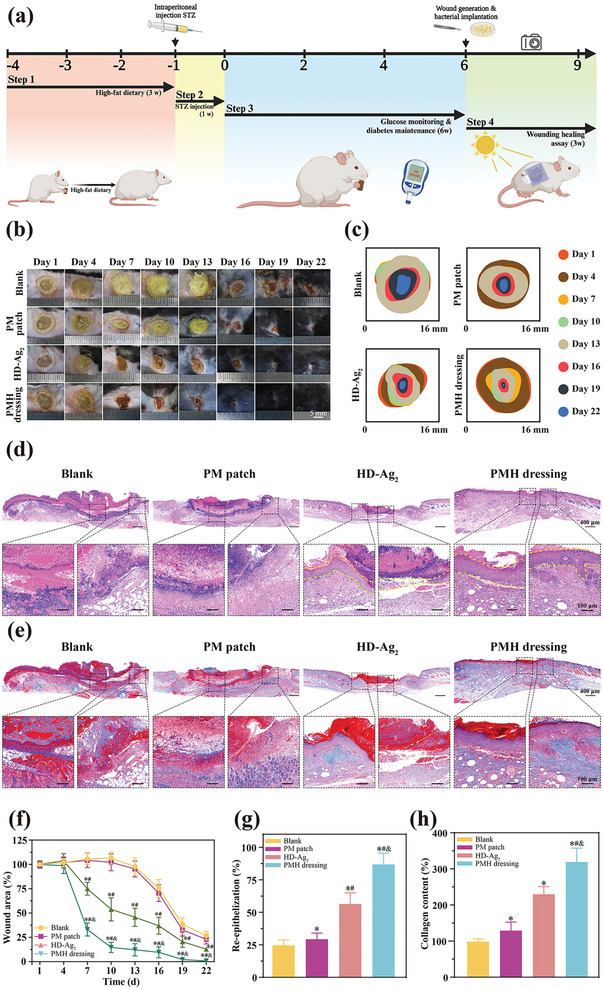
PMH dressing enhanced bacteria‐infected diabetic wound healing in vivo. a) Timeline of model establishment, dressing treatment, wound observation, and tissue harvest in the animal experiment. b) Representative images of the wound at predetermined time points. c) Wound healing boundaries overlayed on each image. d) HE and e) Masson staining of tissue sections from the wounded areas and adjacent skin. f) Analysis of wound closure. g) Analysis of re‐epithelization percentage. h) Analysis of collagen content. Data were shown as mean ± SD (*n* = 3, *p < 0.05 when compared with Blank group; #*p* < 0.05 when compared with PM patch group; and &*p* < 0.05 when compared with HD‐Ag_2_ group).

This study aimed to evaluate the effectiveness of PMH dressings on skin wounds co‐infected with MRSA and PAO. We established type II diabetic mouse models through a high‐fat diet induction, STZ injection, and maintenance of a hyperglycemic state (**Figure** **4**a). In addition, we tested photovoltaic microcurrent patches (referred to as PM patches, which lacked hydrogel compared to PMH dressing) and HD‐Ag_2_ hydrogel. The wounds in the Blank group were treated with a Tegaderm film dressing, a commercially available wound dressing known for its outstanding water repellency and moisture vapor penetrability.^[^ ^55^ ^]^ As shown in Figure 4b, a yellowish purulent discharge was evident around the wounds on day 13 for both the Blank and PM patch groups. In contrast, the yellow exudate disappeared earlier in the PMH dressing group and the HD‐Ag_2_ group (by day 7). On day 22, wounds treated with PMH dressing achieved a high closure ratio of 97%; while, those in the other groups remained unhealed (less than 80%) (Figure 4c,f).

The analysis of wound area demonstrated that both HD‐Ag_2_ and PMH dressing significantly accelerated the wound healing process, with the latter exhibiting the most pronounced therapeutic effect. The role of HD‐Ag_2_ hydrogel in promoting wound healing was especially noteworthy. In environments with ample lighting conditions, OPV cells enabled the PMH dressing to deliver stable and continuous biomimetic ES to the wound. Even under low‐light conditions, the HD‐Ag_2_ hydrogel interface continued to offer therapeutic benefits, making the dressing adaptable for various environments. In addition, we harvested wound tissues at the endpoint of this study and conducted histological analysis through H&E and Masson's staining. Compared with the Blank group, wounds treated with PMH dressing demonstrated a significant reduction in inflammatory cell infiltration, as indicated by the small blue‐stained cells (Figure 4d,e). Moreover, a fundamental epithelial structure was identified at the wound sites after PMH dressing treatment (Figure 4d,g). The results from Masson's staining revealed a significant increase in the regeneration of mature fibrous tissue (identified by intense blue fibers) in the PMH dressing group, with the area of collagen deposition scaling up to ≈320% (with the Blank group normalized to 100%) (Figure 4e,h). Compared with the HD‐Ag_2_ group, the PMH dressing delivered biomimetic ES stimulation to the wound through wearable flexible OPV cells, significantly promoting the healing of wounds in the repair phase. Unlike the recently reported bioelectrical dressing,^[^ ^9^ ^]^ the PMH dressing effectively supplied biomimetic exogenous ES with appropriate electric field strength through seamless contact with the wound via the hydrogel interface, thereby promoting the healing of diabetic wounds.

Skin wounds in diabetic mice, due to their exposure to air and elevated local glucose concentration, are susceptible to bacterial proliferation.^[^ ^56^ ^]^ The HD‐Ag_2_ hydrogel, acting as a tissue–electrode interface, effectively eradicates bacteria present in diabetic wounds. It accelerates the transition from the inflammatory to the repair stage, thereby achieving outstanding therapeutic results. Moreover, chronic inflammation is a hallmark of non‐healing diabetic wounds.^[^ ^57^ ^]^ In diabetic wounds, the pro‐inflammatory to anti‐inflammatory macrophage phenotypic switch is impaired and contributes to chronic inflammation.^[^ ^58^ ^]^ In this context, macrophages mainly exhibit a pro‐inflammatory M1 phenotype and express high levels of inflammatory cytokines (e.g., TNF‐α and IL‐1β); while, the number of M2 phenotype macrophages, which produce anti‐inflammatory and angiogenic cytokines (e.g., TGF‐β_1_ and IL‐10), decreases. In this study, macrophage phenotypes in the wound area were evaluated using iNOS (M1) and TGF‐β1 (M2) immunofluorescence staining. We discovered that during the mid‐healing period (15d), wounds treated with HD‐Ag_2_ and PMH dressing had fewer M1 type macrophages and more M2 type macrophages than the Blank and PM patch groups. Further, we assessed the transcription levels of inflammatory cytokines (TNF‐α, IL‐1β) and anti‐inflammatory cytokines (TGF‐β1, IL‐10) in wound tissues via RT‐qPCR. The results suggest that inflammation levels remained high in the Blank and PM patch groups; while, the HD‐Ag_2_ and PMH dressing groups started transitioning into the repair phase (details can be found in Figure S12, Supporting Information). This can be attributed to the immunomodulatory and anti‐inflammatory activities of high molecular weight (100–250 kDa) hyaluronic acid.^[^ ^59^ ^]^ Hence, the antimicrobial and inflammation‐regulating properties of HD‐Ag_2_ hydrogel in PMH dressing are highly beneficial for the healing of diabetic wounds.

### 2.5 Potential Mechanism of PMH Dressing Affecting Diabetic Wound Healing

**Figure 5:**
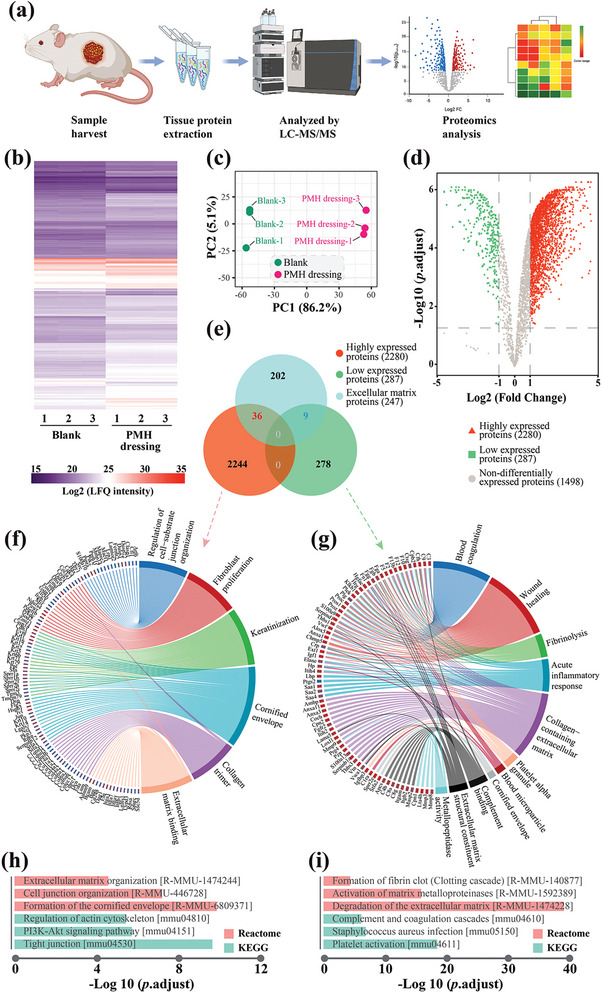
a) Schematic diagram of tissue collection and proteomics analysis. b) The expression levels of identified co‐expressed proteins are illustrated in a heatmap. c) PCA results. d) DEPs are presented in a volcano plot. e) Venn diagram comparing DEPs to an extracellular matrix proteomics dataset. f) Results of the gene ontology (GO) analysis for the upregulated proteins. g) Results of the GO analysis for the downregulated proteins. h) Functional analysis (KEGG and Reactome analysis) results of the upregulated proteins. i) Functional analysis results of the downregulated proteins.

The differential expression of proteins (DEPs) between the Blank group and the PMH dressing group was identified through label‐free proteomics (LFP) (**Figure** **5**a). In total, 4066 proteins were identified as co‐expressed through LFP detection (Figure 5b). Principal component analysis (PCA) demonstrated markedly different component profiles between the two groups (Figure 5c). In the PMH dressing group, 2280 proteins were upregulated (with a *p* value < 0.05 and a fold change value greater than 2); while, 287 proteins were downregulated (with a *p* value < 0.05 and a fold change value less than 0.5) (Figure 5d). The results of the GO enrichment analysis showed that proteins related to extracellular matrix collagen deposition and the keratinization of epithelial cells were significantly upregulated (Figure 5f). In contrast, downregulated proteins were enriched in coagulation, inflammatory response, extracellular matrix, and matrix metalloproteinase (MMP) activity (Figure 5g). The functional enrichment analysis results for DEPs indicated that upregulated proteins participate in extracellular matrix organization, intercellular connections, and the formation of the cornified envelope (Figure 5h). Downregulated proteins were enriched in coagulation, bacterial infection, MMP activity, and degradation of the extracellular matrix (Figure 5i).

**Figure 6:**
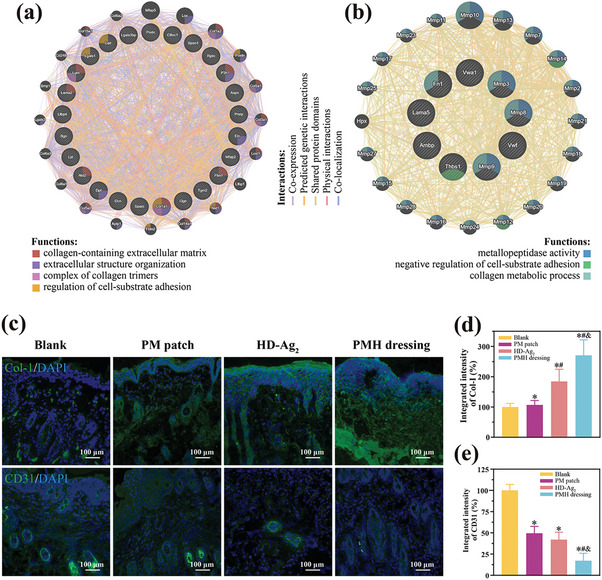
Pathway analysis of the a) highly expressed DEPs and b) low expressed DEPs correlated with extracellular matrix reveals significant enrichment of proteins involved in collagen organization and metallopeptidase activity. GeneMANIA (https://genemania.org/) was used for the analysis. c) Immunofluorescence observation of collagen‐I (Col‐I) and CD31 staining. The immunofluorescence intensity of positive d) Col‐I and e) CD31 cells present at the wound beds in each group was compared to that of the blank group, which was set as 100%, with other groups calculated as a percentage. Data were shown as mean ± SD (*n* = 3, *p < 0.05 when compared with Blank group; #*p* < 0.05 when compared with PM patch group; and &*p* < 0.05 when compared with HD‐Ag_2_ group).

To further understand the effects of PMH dressing treatment on the extracellular matrix, we analyzed the ECM‐related components among the DEPs and identified 36 upregulated and 9 downregulated proteins (Figure 5e). The upregulated proteins included various ECM components, such as collagens, elastin, fibrillin, and laminin. In addition, proteins related to intercellular junctions, such as galectin and spondin, along with proteins associated with keratinocyte differentiation, such as repetin and transglutaminase, were also found to have increased expression (Table S4, Supporting Information). Enzymes associated with ECM degradation, specifically MMP, and proteins involved in the activation of coagulation, such as von Willebrand factor and thrombospondin, were observed to be downregulated (Table S5, Supporting Information). Further, an interaction network analysis of the 45 differential extracellular matrix proteins suggests that PMH dressing treatment promotes collagen deposition (**Figure** **6**a,b). Through immunofluorescence techniques, we studied the state of collagen regeneration and vascular distribution in the tissues of all groups. We noted that wounds treated with PMH dressing exhibited markedly higher collagen deposition than the other wounds, evident from the brighter green fluorescence (Figure 6c,d). At the same time, the quantity of blood vessels (detected through the vascular endothelial‐specific antigen CD31) in wounds treated with PMH dressing was significantly diminished (Figure 6c,e).

ES has been shown to increase the growth, migration, and extracellular matrix synthesis of fibroblasts derived from diabetic patients.^[^ ^60^ ^]^ The combination of ES with wearable devices^[^ ^25^ ^]^ or biodegradable, electroactive hydrogels^[^ ^61^ ^]^ has yielded promising results in regulating skin cell behaviors and accelerating the wound healing process. Herein, the PMH dressing integrates the advantages of wearable devices and electroactive hydrogels. Through the external supply of biomimetic ES, it actively modulates collagen deposition and remodeling; thus, accelerating the healing process. It is also worth noting that proteins related to vascular function are downregulated in wound tissues treated with PMH dressing. The formation of new blood vessels during wound repair is indispensable for efficient tissue repair. Nevertheless, recent studies imply that reducing angiogenesis experimentally may be advantageous to boost long‐term healing results.^[^ ^62^ ^]^ Both fetal skin and oral mucosal wounds demonstrate fast healing with little scarring.^[^ ^63^ ^]^ Moreover, wounds treated with PMH dressing transition to the remodeling phase earlier than those with Tegaderm. This phase entails the reorganization, degradation, and resynthesis of the extracellular matrix; while, granulation tissue is progressively remodeled into acellular and less vascular scar tissue.^[^ ^64^ ^]^

The role of MMPs is to facilitate wound debridement and regulate the metabolism of the ECM during the healing of diabetic wounds.^[^ ^65^ ^]^ The two primary active MMPs in human diabetic wound tissues, MMP‐8 and MMP‐9, have specific roles in wound healing.^[^ ^66^ ^]^ A study by Chang et al. assessed how these two MMPs affect the repair of diabetic wounds in mice, revealing that supplementary MMP‐8 treatment facilitated wound closure, whereas MMP‐9 negatively impacted healing.^[^ ^67^ ^]^ Our study; however, observed a downregulation of both forms of MMPs, which differs from prior findings. To understand this inconsistency, we conducted immunofluorescence staining on wound tissues during the early (7d), middle (15d), and late (22d) healing stages. We observed a high expression of MMP‐8 in wounds treated with PMH dressing during the early and middle stages, decreasing in the later stage, whereas MMP‐9 was mostly expressed early on (Figure S13, Supporting Information). Conversely, in the Blank group, MMP‐8 was mainly expressed in the late stage; while, MMP‐9 was abundant throughout. Our investigation focused on the late healing stage (when the wound is transitioning from repair to remodeling with a low expression of both MMPs), unlike Chang et al.’s study, which focused on the middle stage (high MMP‐8, low MMP‐9). Overall, the proposed PMH dressing could elevate MMP‐8 levels in diabetic wounds, accelerate the transition from repair to remodeling, and promote healing.

Overall, these results suggest that the PMH dressing aids in wound healing by modulating extracellular matrix regeneration, eliminating bacteria, regulating inflammatory responses, and altering vascular functions. Therefore, the PMH dressing not only emerges as a therapeutic intervention but also enhances our understanding and strategy for diabetic wound care.

## 3 Conclusion

In this study, we developed the PMH dressing, a novel sandwich‐structured solution tailored to address the intricate challenges of diabetic wound care. By synergistically integrating flexible OPV cells, MEMS electrodes, and the multifunctional HD‐Ag hydrogel, we designed a wireless bioelectrical wound dressing that not only delivers biomimetic ES but also ensures optimal tissue–electrode interfacing. Leveraging ultraviolet light, we synthesized the HD‐Ag hydrogel, which serves as a tissue‐electrode interface for ES delivery. Our findings confirmed the hydrogel's superior biocompatibility and antibacterial activity against common pathogenic bacteria. The dressing exhibited significant therapeutic effects on diabetic wounds complicated by bacterial infections. We demonstrated that the PMH dressing promotes the healing of diabetic wounds by regulating the regeneration and remodeling of extracellular matrix proteins, accelerating re‐epithelialization, and modulating chronic inflammation. As a groundbreaking prototype that integrates organic OPV, MEMS microcurrent electrodes, and hydrogel, the PMH dressing has the potential to usher in a new generation of more effective and patient‐friendly wound care solutions. While our research has confirmed the benefits of PMH dressing on diabetic wounds, there is insufficient evidence supporting its effectiveness on other prevalent chronic wounds such as burns, pressure ulcers, and radiation skin injuries, presenting a research direction for future studies. In addition, our forthcoming study will focus on the development of environmentally sustainable and recyclable wound dressings that are also cost‐effective; thus, setting the stage for potential industrialization.

## 4 Experimental Section

### Fabrication and Characterization of Flexible OPV Cells

PM6 and Y6, purchased from Chemscitech Inc. (Canada), were used as received. Poly(2‐methoxy‐5‐(3′,7′‐dimethyloctyloxy)−1,4‐phenylenevinylene) (PM6), a conjugated polymer, had been employed to develop efficient, low‐cost, and lightweight OPV cells. In addition, another conjugated polymer, known as Y6 Polymer, was also utilized in the development of such OPV cells. The molecular formulas of PM6 and Y6 are shown in Figure S1a,b, Supporting Information. In this study, the authors introduced a (TPE) to enhance the mechanical properties such as stable interfacial adhesion and bending durability and to further improve the optoelectronic properties (Figure S1c, Supporting Information). All supplies were used as received. ITO glass, with a sheet resistance of 15 Ω sq^−1^, was purchased from CSG HOLDING Co., Ltd. (China). ZnO nanoparticles used in this layer were synthesized following a reported protocol.^[^ ^68^ ^]^ Active layer solutions were prepared by mixing PM6 and Y6 materials with o‐xylene and 1,8‐diiodooctane (DIO) in a specific ratio (D/A ratio of 1:1.2). These solutions were spin‐coated onto the substrate in an N_2_ glove box to form the active layers of the OPV cells. After applying the active layers, a thin layer of MoO*x* was deposited as an anode interlayer via vacuum deposition. This layer helped improve the performance of the solar cells by facilitating electron transfer from the active layers to the Ag top electrode. The Ag electrode was then deposited onto the device at a thickness of 100 nm, completing the fabrication of the OPV cells.

Fabrication of large‐area and module OPV cells was conducted under ambient conditions instead of in a controlled environment like an N_2_ glove box. The device fabrication method was adapted from the published work with appropriate modifications.^[^ ^69^ ^]^ The bottom electrode of the cells was made from PET/silver‐grid substrates with a striped pattern, which had a sheet resistance of 10 Ω sq^−1^ and a transmittance of 95% or higher. The R2R instrument (MiniRoll Coater, FOM Technologies) was used to fabricate the large‐area flexible OPV cells. Modules with two and four serial stripes were made using this process. After optimization, ZnO nanoparticles dispersed in isopropanol were selected to form the electron transport layer of the devices. The concentration of the ZnO nanoparticles was maintained at 15 mg mL^−1^. The active layers of the OPV cells were produced by dissolving the donors and acceptors in o‐xylene, with DIO added as an additive solvent. The active layer solution was then applied using a slot‐die coater. After applying the active layers, the devices were placed in a vacuum evaporation system to deposit a thin layer of MoO*x* as the anode interlayer and a layer of Ag as the top electrode. The active areas of the flexible OPV modules were 1.25 and 2.50 cm^2^, respectively. The anode made contact with the electrode on the MEMS electrode; while, the cathode was designed to connect with the skin. The PEDOT: PSS material (Clevios PH1000, Heraeus, Germany) was spin‐coated onto the PET/Ag‐grid substrates at 800 rpm and then baked in ambient air (120 °C for 15 min). This process enhanced the surface flatness and wettability of the PET/Ag‐grid substrates, making them suitable for use as the bottom electrode of the OPV cells.

The fabrication process for the rest of the OPV cells was identical to that used for the reference cells made on glass/ITO substrates. This involved applying the ZnO electron transport layer, the active layers, and the anode interlayer and top electrode using the methods described above.^[^ ^35^, ^38^ ^]^ These steps completed the fabrication of the OPV cells on PET/Ag‐grid/PH1000 hybrid electrodes. The OPV cells were also fabricated with patterned ITO glass, a transparent conductive material used as the substrate for the active layers. After cleaning the ITO glass, a ZnO electron transport layer was prepared by spin‐coating at 3000 rpm. This layer assisted in transporting electrons generated by the active layers to the electrodes, where they could be collected and used to generate electricity.

The electrical output of the device was evaluated under AM 1.5G (100 mW cm^−2^) conditions with a Newport Thermal Oriel 91159A solar simulator, and mechanical modeling was conducted using finite element simulation tools.

### Fabrication and Characterization of Flexible MEMS Electrodes

The fabrication of flexible MEMS electrodes involved several steps. The first step entailed depositing a layer of UV photoresist onto a glass substrate and patterning it using photolithography. This process rendered a specific shape, such as a hexagonal honeycomb structure, on the substrate. This shape promoted maximum light transmission and exhibited low optical loss, resulting in a high light transmittance (80–90%) in the flexible electrode. The subsequent step involved patterning the PET substrate. This was achieved by applying UV glue to the PET substrate and imprinting the patterned UV photoresist film atop the UV glue using a nickel master. This action created a mask‐like pattern on the grid, reserving a grid thickness of 3 µm. The third step consisted of filling the grooves in the patterned PET substrate with silver nano ink and sintering the ink at 150 °C for 15 min. This process formed a conductive silver grid on the PET substrate. The grid was then electroplated with copper for ≈5 min, utilizing an electroplating current of 2 A. This allowed for a dense copper coating that effectively prevented further oxidation. Lastly, the surface of the film was smoothed with an aqueous solution containing silica particles. This action created a dense microstructure on the surface of the Cu layer, which passivated the electrodes and prevented further oxidation. These steps concluded the fabrication of the flexible MEMS electrodes.

The structure of the microcircuit on the MEMS electrode was examined using a scanning electron microscope (SEM, SU‐70, Hitachi, Japan), and mechanics modeling was conducted using finite element simulation tools.

### Biocompatibility of Flexible OPV Cells and Flexible MEMS Electrodes

HaCaT were employed to test the cellular biosafety of flexible OPV cells and MEMS electrodes. HaCaT cells were cultivated using a standard protocol.^[^ ^70^ ^]^ The electronic devices were disinfected by immersion in 75% alcohol for 30 min; and then, equilibrated in the culture medium for 12 h. Cells were seeded in 12‐well plates at a density of 50 000 cells per well and co‐cultured with the electronic device samples (25 mg mL^−1^). After 1 and 3 days of co‐culturing, cell morphology was observed under an optical microscope. Cell viability at predetermined time points was assessed using a Live/Dead staining kit and a CCK‐8 cell viability assay kit. The stained cells were observed under an inverted fluorescence microscope (IX53, Olympus).

The extracts of the electronics were prepared for the hemolysis experiment. Flexible OPV cells and MEMS electrode samples were immersed in DMEM culture medium (25 mg mL^−1^) and incubated at 37 °C for 48 h to prepare the conditioned medium. Erythrocytes were separated from rat blood by centrifugation (120 × *g*, 10 min) and washed three times with DPBS (resuspending and centrifuging each time) to remove damaged erythrocytes. The methodology for the hemolysis test was consistent with the procedure detailed in the published works.^[^ ^71^ ^]^ Triton X‐100 (0.1%) was used as the positive control; while, DPBS served as the blank control. The hemolysis rate (H) was calculated according to Equation (1):

(1) $$H=\frac{A_{p}-A_{b}}{A_{t}-A_{b}}\times 100\%$$

where *A*_p_ represents the absorbance of the test electronic device sample, *A*_t_ represents the absorbance of the positive control (Triton X‐100), and *A*_b_ represents the absorbance of the blank control (DPBS).

### Synthesis of HAMA and Dopamine Grafting

Methacrylic anhydride (MA), 2‐hydroxy‐4′‐(2‐hydroxyethoxy)−2‐methylpropiophenone (I2959), *N*‐(3‐dimethylaminopropyl)‐*N*′‐ethylcarbodiimide hydrochloride (EDC), hydroxyl‐PEG‐NHS ester (NHS), and Irgacure 2959 (I2959) were procured from Aladdin Chemistry Co., Ltd (Shanghai, China). Sodium hyaluronate (≈1.8–2.2 MDa) was sourced from Bloomage Biotechnology Co., Ltd (Shandong, China). The synthesis of HAMA followed a previously reported method with minor modifications.^[^ ^72^ ^]^ Briefly, hyaluronate (HA; 3 g, monomer molar amount = 8 mmol) was dissolved in deionized water (300 mL) and stirred for 2 h to form a 1 wt% solution. Methacrylic anhydride (MA, 12 mL, 80 mmol) was then gradually added to the mixture. The solution was placed in an ice bath and vigorously stirred using a digital overhead stirrer (Eurostar 20 Digital, IKA). The pH of the mixture was monitored and, if necessary, adjusted to 8.5 with a 0.1 m NaOH solution. The reaction was allowed to proceed for 24 h. The product was dialyzed for 2 days using a membrane with a molecular weight cutoff (MWCO) of 14 000 Da against deionized water, which was refreshed three times daily to remove unreacted reagents and byproducts. Finally, the product was lyophilized and stored at −20 °C until use.

Dopamine (DA) was grafted onto HAMA using EDC as a crosslinker, following a previous study.^[^ ^73^ ^]^ HAMA (260 mg, monomer molar amount = 0.5 mmol) was dissolved in 10 mL deionized water and stirred for 24 h at room temperature. EDC (115 mg, 0.6 mmol) and NHS (69 mg, 0.6 mmol) were added to this solution. After 20 min, DA (114 mg, 0.6 mmol) was added, and the pH of the mixture was adjusted to 5.5 via the addition of HCl (0.1 m) and NaOH (0.1 m). The mixture reacted overnight under nitrogen at room temperature. Finally, the solution was dialyzed against deionized water using a membrane (MWCO < 14 000 Da) for 7 days under acidic conditions. The product, HAMA‐DA, was then lyophilized and stored at −20 °C until use.

The chemistry of HD was analyzed using the ^1^H‐NMR spectrum, and the catechol content was quantified by measuring the optical absorbance at 280 nm with a spectrometer (UV‐1800, Shimadzu). A dopamine standard curve was generated for quantification of DA, which indicated that the degree of dopamine substitution for HD was ≈10%.

### Preparation of HD‐Ag Hydrogels

HD was dissolved in a phosphate buffer solution (PBS) to form a 2 wt% solution. Silver nitrate (AgNO_3_) was added to the solution at varying concentrations of 1, 2, and 3 mg mL^−1^. The photoinitiator I2959 (0.5 wt%) was then incorporated under vigorous stirring. Subsequently, these hydrogel precursors were pipetted onto substrates and exposed to ultraviolet radiation (365 nm, 300 mW cm^−2^) for 1 min to complete gelation using a UV lamp (Lumen Dynamics Group Inc., Canada). The resulting hydrogels were labeled as HD‐Ag_1_, HD‐Ag_2_, and HD‐Ag_3_, respectively, where HD denotes hydrogels without silver content.

### Characterizations of HD‐Ag Hydrogels

Several tests were conducted to investigate the morphological and physicochemical characterizations of HD and HD‐Ag hydrogels. These included observations under SEM and transmission electron microscopy (TEM), X‐ray photoelectron spectroscopy (XPS) analysis, rheological tests, Ag^+^ ion release studies, conductive tests, and swelling tests. The hydrogels were dried using a vacuum freeze‐drier (Scientz‐18ND, Ningbo, China); then, sputter‐coated with a thin gold layer for SEM observation (S‐4800, Hitachi, Japan). For TEM investigation, the hydrogel samples were fixed in a mixture of 4% paraformaldehyde and 2% glutaraldehyde in 0.1 m sodium cacodylate (pH 7.4). After rinsing with cacodylate (0.1 m), the samples were post‐fixed with 1% osmium tetroxide to enhance contrast and then dehydrated through a graded series of ethanol solutions. Subsequently, the samples were embedded in resin, sectioned, mounted on TEM grids, and stained with uranyl acetate and lead citrate. Images were acquired at an accelerating voltage of 80 kV using a TEM. XPS analysis was conducted using an ESCALAB 250Xi spectrometer (ThermoFisher Scientific, USA) in the range of ≈0–1200 eV to determine the valence state of silver within the hydrogel system. For this, HD and HD‐Ag_2_ were tested as examples of pure hydrogel and silver‐doped hydrogel, respectively.

The rheological behavior and photocuring kinetics of the samples were examined using a rheometer (Discovery hybrid rheometer 2, USA) with an LED accessory emitting light at 365 nm.^[^ ^74^ ^]^ The release of in vitro Ag^+^ ions was quantified by immersing the hydrogel samples in phosphate‐buffered saline (PBS, 20 µL µg^−1^) at 37 °C. At predetermined time points (1, 2, 6, 24, 48, 72, and 96 h), the supernatant was collected and the media were replenished with an equal volume of fresh PBS. The released Ag^+^ ion concentration in the supernatant was measured by a spectrophotometer (Shimadzu, Japan) based on the formation of a ternary complex among the Ag^+^ ion, phenanthroline, and eosin Y in acidic aqueous media (pH 6, adjusted by dilute NaOH and HNO_3_ solutions).^[^ ^75^ ^]^ The absorbance was measured at the wavelength of 550 nm. The concentration of Ag^+^ was calculated based on a calibration curve prepared by silver ion standard solutions ranging from 0 to 0.2 mg L^−1^. All experiments were carried out in triplicate.

The electrical conductivities (*σ*) of the hydrogels were determined using an electrochemical workstation (CHI660e, CH Instruments, Shanghai, China). The hydrogel samples were prepared as discs with a bottom area of 66.5 mm^2^ and a height of 3 mm. The electrical conductivities were measured at a frequency of 105 Hz and magnitude of 0.5 V at open circuit voltage in accordance with Equation (2).

(2) $$\sigma =\frac{L}{R\times S}$$

where *L* indicates the height, *S* indicates the bottom area of the hydrogel sample, and *R* denotes its bulk resistance.

To intuitively compare the conductivity differences among the hydrogels of each group, a parallel circuit was constructed that included an LED indicator and a test bench for gel samples. The test bench consisted of 1.5 mL EP tubes connected to conductive copper foil onto which the hydrogel was polymerized using ultraviolet irradiation. Once the external power supply was activated, the variations in the brightness of the LED indicated the differences in the electrical conductivities of the hydrogels. The swelling ratio (SR) of the HD‐Ag hydrogels was calculated using the mass change between the swollen and dry hydrogel. Briefly, lyophilized hydrogel samples were immersed (≈30 mg) in PBS (2 mL) and incubated at 37 °C. At pre‐determined time intervals (0, 1, 6, 12, 24, 36, 48, 60, and 72 h), the samples were removed from the tubes. The surface buffer was then carefully removed using filter paper and the swollen samples were weighed. The swelling ratio was calculated as SR = (*W*_t_ – *W*_d_)/*W*_d_ × 100%, where *W*_d_ represents the weight of the dry hydrogels and *W*_t_ represents the weight of the swollen hydrogels at different time points. Three independent samples were weighed from each group.

### Antibacterial Evaluation of the Hydrogels

The antibacterial activities of the HD‐Ag hydrogels were evaluated using the inhibition zone assay and colony count method. For this study, MRSA and PAO were cultured in Luria–Bertani (LB) broth (Difco, Becton Dickinson GmbH, Germany) at 37 °C in an aerobic environment. Both types of bacteria were quantified by measuring the optical density at 600 nm with a spectrophotometer, according to the established standard curve. The antibacterial property of the HD‐Ag hydrogels was evaluated using the inhibition zone method. Briefly, MRSA or PAO was sprayed in PBS (1 × 10^8^ CFU mL^−1^, 50 µL) on 6‐cm‐diameter LB agar plates. Disc‐shaped hydrogel samples (5 mm in diameter and 0.5 mm thick) were then placed in the central region of these agar plates. After 12‐h incubation at 37 °C, the agar plates were photographed, and the area of the clear zone around the hydrogel samples was measured using Image J software (National Institute of Health, USA).

The antibacterial activity of the HD‐Ag hydrogels was further investigated through the colony count assay. In brief, MRSA or PAO (500 µL of 1 × 10^8^ CFU mL^−1^) was co‐cultured with HD‐Ag hydrogel samples (5 mm in diameter and 0.5 mm thick) in a 24‐well plate at 37 °C under aerobic conditions in LB medium for 12 h. The culture media was then serially diluted, seeded them on corresponding LB agar plates, and allowed to grow. Following a further period of growth (12 h), the plates were photographed, and the antibacterial properties of each hydrogel sample were assessed by counting the number of colonies formed.

### Cytocompatibility Evaluation of the HD‐Ag Hydrogels

NIH/3T3 and HaCaT cells were selected to study the biocompatibility of the HD‐Ag hydrogels. Both cell types were purchased from Procell Life Science&Technology Co., Ltd (Wuhan, China) and cultured following the same protocol as in the literature.^[^ ^70^ ^]^ Precursor solutions (150 µL) from each group were used to fabricate hydrogel disks (8 mm in diameter and 3 mm in height) through photo‐crosslinking gelation. After sterilizing the disks in 75% alcohol and equilibrating them in culture medium, the cells were seeded at a density of 1 × 10^4^ cells per well into 24‐well plates. After an overnight cultivation, the hydrogel disks were added to each well, ensuring the level of culture medium was slightly lower than the upper surface of the disk to facilitate cell‐hydrogel contact. The cells were observed after 1, 3, and 5 days of incubation. Post incubation, DMEM containing 10% CCK‐8 reagent was added after removing both the disk and medium from each well. The absorbance was measured at 450 nm with a microplate reader (Multiscan; Thermo Scientific, USA) after transferring the medium (100 µL) from each well into a new 96‐well plate. The tissue culture polystyrene without the hydrogel served as the control. In addition, the cells were stained with Calcium AM (4 µm) and EthD‐1 (2 µm) in PBS for 30 min after their incubation with hydrogels for 3 days, following the instructions provided in the live/dead viability kit (Life Technologies, USA). Three random fluorescence images were acquired from each sample, and this process was repeated for three independent experiments.

### Establishment of Type II Diabetic Mice Model

The protocol of the animal experiment was reviewed and approved by the Animal Ethics and Welfare Committee (AEWC) of Ningbo University. Approval from the institutional animal ethics committee was obtained before conducting the animal experiment (approval number: NBU20230142). C57BL/J mice (6‐week old, male) were purchased from Beijing Vital River Laboratory Animal Technology Co., Ltd. and housed in plastic cages with a 12‐h light/dark cycle in a specific pathogen‐free environment. The temperature and humidity were maintained at 25 °C and 60%, respectively. The mice were continuously fed a high‐fat diet (60% of calories from fat, XTHF60, Jiangsu Xietong Medicine Bioengineering Co., Ltd., Jiangsu, China) for 1 month. The mice were rendered diabetic by injection of streptozotocin (STZ). Specifically, the mice were fasted for 4 h; and then, intraperitoneally injected with STZ (40 mg kg^−1^ dissolved in 50 mm sodium citrate buffer, pH 4.5) for 5 consecutive days. Glucose solution (5%, 1 mL) was injected intraperitoneally 6 h after STZ treatment to prevent fatal hypoglycemia. In addition, sucrose water (10%) was routinely provided. The body weights of the mice were monitored weekly. Daily blood glucose levels in fasting mice (fasting starting at 7 a.m., blood drawn between 1 and 3 p.m.) were measured using a One‐Touch Ultra glucometer (Lifescan Inc.) after STZ administration. Mice with fasting blood glucose levels above 300 mg dl**^−1^** (or 16.7 mm) were identified as diabetic.^[^ ^76^ ^]^ Diabetes was maintained for 4 weeks to permit manifestation of disease‐related changes. During this period, the diabetic mice were given subcutaneous insulin injections (0.5 U, recombinant human insulin, Beijing Solarbio Science & Technology Co.) on alternate days to ensure their survival. Thus far, the type II diabetic animal model was successfully established.

### Creation and Treatment of Bacteria‐Infected Diabetic Wounds

The mice were anesthetized using intraperitoneal injection with a combination of Zoletil 50 (30 mg kg^−1^, Virbac, France) and xylazine hydrochloride (10 mg kg^−1^, Huamu Animal Health Products Co., Ltd., China). The mice's dorsal fur was removed with an electric clipper. Additional hair removal was carried out using a non‐irritating depilatory cream to fully expose the surgical area. Subsequently, a circular 10‐mm‐diameter wound was created on the back of each mouse. Then, a bacterial suspension blend of MRSA (25 µL, 1 × 10^7^ CFU mL^−1^) and PAO (25 µL, 1 × 10^7^ CFU mL^−1^) was sprayed onto the wound and covered with a TegadermTM film. Four kinds of wound dressings were prepared: a) Tegaderm film dressing (Blank), b) flexible photovoltaic MEMS electrode (PM patch), c) HD‐Ag_2_ hydrogel, and d) PMH dressing. Simulated indoor light (2700K, 1000 lux) (Enlitech, Kaohsiung city, Taiwan) was applied as the illumination for the OPV cells. Mice treated with PM patch and PMH dressing were exposed to this light for 2 h daily. All the dressings were replaced, and images of the wounds were captured every 3 days. At the end of the study, mice were asphyxiation with CO_2_ asphyxiation, and tissue samples were harvested for subsequent tests.

### Histological Analysis

The wound tissues were fixed, dehydrated, paraffin‐embedded, and sectioned, following the same protocol as in the literature.^[^ ^77^ ^]^ The tissue samples were stained according to the instruction of hematoxylin & eosin (H&E) and Masson kit (Solarbio Science & Technology, Beijing, China).

### Quantitative Label‐Free Global Proteomic Analysis

A total of 100 mg of general protein was extracted from wound tissue treated with Tegaderm film (Blank group) and PMH dressing, the methodology of which followed the previously published work.^[^ ^78^ ^]^ The skin tissue samples were homogenized and centrifuged at 10000 × *g* for 5 min. The supernatant, containing the general protein extract was then collected, and the protein concentration was measured by BCA method.^[^ ^79^ ^]^ Following this, the proteins were denatured at 100 °C for 15 min. Next, the protein sample (100 µg) was diluted in a solution containing 50 mm ammonium bicarbonate buffer (200 µL). To reduce any disulfide bonds present within the primary structure of protein, dithiothreitol (DTT; 5 mm; Solarbio Bioscience & Technology Co., Ltd; Shanghai China) was added and allowed to react at 37 °C for 2.5 h. This was followed by alkylation with iodoacetamide (IAA, 500 mm) for 40 min under dark conditions at 37 °C. The resultant mixture was digested by adding a trypsin solution (25 µL, 1 µg µL^−1^, Sigma‐Aldrich, USA) and incubated at 37 °C for 16 h.

Before conducting a nano‐flow LC‐MS/MS analysis of the digested peptides, the samples were diluted tenfold with 0.1% formic acid (Sigma–Aldrich) in an aqueous solution, resulting in an estimated peptide concentration of 0.2 µg µL^−1^. The analysis protocol for peptide samples was similar to the work of Tang and colleagues.^[^ ^80^ ^]^ The mass spectrometry proteomics data have been deposited to the ProteomeXchange Consortium via the iProX partner repository^[^ ^81^ ^]^ with the dataset identifier PXD044920.

Proteomic analysis was performed using the freely available R language‐based platform Wu Kong (https://wkomics.omicsolution.com/wkomics/main/). Protein intensities from samples in the Blank and PMH dressing groups were extracted from Proteome Discoverer result files to represent their respective expression levels. Proteins with more than 50% missing LFQ intensity across all samples were filtered out, and a normalization to the median along with a k‐nearest neighbors (KNN) imputation was executed to generate the missing values. For more precise quantitation results, proteins exhibiting an intensity coefficient of variation larger than 0.3 were further filtered. A 4065 × 6 protein expression matrix was then generated for subsequent statistical analysis. An unpaired Student's *t*‐test was performed to identify differentially expressed proteins (DEPs) between the Blank and PMH dressing groups, using a Benjamini–Hochberg (BH) adjusted *p* value < 0.05 and a fold change > 2.0 or < 0.5 for upregulation or downregulation, respectively. The biological function of the DEPs was analyzed through GO, KEGG, and Reactome pathway‐enrichment analysis.^[^ ^82^ ^]^ In addition, a comparison between DEPs and an extracellular matrix protein dataset (KW‐0272) downloaded from the Uniprot database (https://www.uniprot.org/) identified overlapping proteins. These results were illustrated using a Venn diagram.^[^ ^83^ ^]^ Further, an interaction network comprising co‐expression, predicted genetic interactions, shared protein domains, physical interactions, and co‐localization among those overlapping proteins, was constructed through GeneMANIA (https://genemania.org/).

### Immunofluorescence Staining

Tissue slices were individually stained with primary antibodies (anti‐Col‐I, anti‐CD31, anti‐iNOS, anti‐TGF‐β1, anti‐MMP‐8, and anti‐MMP‐9, all obtained from Proteintech, Wuhan, China). After thorough rinsing, the slices were treated with secondary antibodies labeled with FITC (green fluorescence) or TRITC (red fluorescence) for color visualization. The nuclei were stained with a 4′,6‐diamidino‐2‐phenylindole (DAPI) containing mounting solution. Slides were then observed under an inverted fluorescence microscope (IX53, Olympus).

### Real‐Time Quantitative Polymerase Chain Reaction (RT‐qPCR)

Tissue samples were crushed and ground at low temperatures. The protocol for total RNA extraction and reverse transcription was the same as in other literature,^[^ ^84^ ^]^ where total RNA (2 µg) was added to a 40 µL reverse transcription reaction system. Relative mRNA expression levels were detected by RT‐qPCR on the LightCycler 96 instrument (Roche Diagnostics, USA). The primer sequences for RT‐qPCR, obtained from the Shanghai Shenggong Biotech Co., Ltd, are as follows:

TNF‐α (Fwd: CCTGTAGCCCACGTCGTAG, Rev: GGGAGTAGACAAGGTACAACCC), IL‐1β (Fwd: GCAACTGTTCCTGAACTCAACT, Rev: ATCTTTTGGGGTCCGTCAACT), TGF‐β1 (Fwd: CCACCTGCAAGACCATCGAC, Rev: CTGGCGAGCCTTAGTTTGGAC), and IL‐10 (Fwd: GCTCTTACTGACTGGCATGAG, Rev: CGCAGCTCTAGGAGCATGTG).

### Statistical Analysis

All quantitative data were presented as mean ± standard deviation (SD). For normally distributed data sets with equal variances, one‐way ANOVA testing followed by a Tukey post‐hoc test was carried out across groups. Statistical analysis was carried out using GraphPad Prism Software (GraphPad Prism, CA, USA). In all cases, significance was defined as *p* < 0.05. The sample sizes (*n*) were no less than three.

## Conflict of Interest

The authors declare no conflict of interest.

## Supporting information

Supporting Information

## Data Availability

The data that support the findings of this study are available from the corresponding author upon reasonable request.

## References

[1] A. D. Lopez , C. D. Mathers , M. Ezzati , D. T. Jamison , C. J. Murray , Lancet 2006, 367, 1747. 16731270 10.1016/S0140-6736(06)68770-9

[2] L. Mude , B. K. R. Sanapalli , A. N. V , S. K. Singh , V. V. S. R. Karri , Drug Dev. Res. 2021, 82, 503. 33432634 10.1002/ddr.21788

[3] a) J. R. Bardill , M. R. Laughter , M. Stager , K. W. Liechty , M. D. Krebs , C. Zgheib , Acta Biomater. 2022, 138, 73; 34728428 10.1016/j.actbio.2021.10.045 PMC8738150

[4] B. Reid , M. Zhao , Adv. Wound Care 2014, 3, 184. 10.1089/wound.2013.0442 PMC3928722 24761358

[5] a) R. Luo , J. Dai , J. Zhang , Z. Li , Adv. Healthcare Mater. 2021, 10, e2100557; 10.1002/adhm.202100557 33945225

[6] S. B. Rajendran , K. Challen , K. L. Wright , J. G. Hardy , J. Funct. Biomater. 2021, 12, 40. 34205317 10.3390/jfb12020040 PMC8293212

[7] A. Zulbaran‐Rojas , C. Park , N. El‐Refaei , B. Lepow , B. Najafi , J. Diabetes Sci. Technol. 2023, 17, 15. 34328024 10.1177/19322968211035128 PMC9846397

[8] M. Ashrafi , T. Alonso‐Rasgado , M. Baguneid , A. Bayat , Exp. Dermatol. 2017, 26, 171. 27576070 10.1111/exd.13179

[9] a) Y. Liang , H. Tian , J. Liu , Y. Lv , Y. Wang , J. Zhang , Y. Huang , Bioelectrochemistry 2020, 135, 107578; 32534380 10.1016/j.bioelechem.2020.107578

[10] a) Y. Jiang , A. A. Trotsyuk , S. Niu , D. Henn , K. Chen , C.‐C. Shih , M. R. Larson , A. M. Mermin‐Bunnell , S. Mittal , J.‐C. Lai , A. Saberi , E. Beard , S. Jing , D. Zhong , S. R. Steele , K. Sun , T. Jain , E. Zhao , C. R. Neimeth , W. G. Viana , J. Tang , D. Sivaraj , J. Padmanabhan , M. Rodrigues , D. P. Perrault , A. Chattopadhyay , Z. N. Maan , M. C. Leeolou , C. A. Bonham , S. H. Kwon , et al., Nat. Biotechnol. 2022, 41, 652; 36424488 10.1038/s41587-022-01528-3

[11] A. Sharma , V. Panwar , B. Mondal , D. Prasher , M. K. Bera , J. Thomas , A. Kumar , N. Kamboj , D. Mandal , D. Ghosh , Nano Energy 2022, 99, 107419.

[12] a) H. Yuk , B. Lu , X. Zhao , Chem. Soc. Rev. 2019, 48, 1642; 30474663 10.1039/c8cs00595h

[13] a) S. R. Madhvapathy , J.‐J. Wang , H. Wang , M. Patel , A. Chang , X. Zheng , Y. Huang , Z. J. Zhang , L. Gallon , J. A. Rogers , Science 2023, 381, 1105; 37676965 10.1126/science.adh7726

[14] a) J. Y. Oh , Z. Bao , Adv. Sci. 2019, 6, 1900186; 10.1002/advs.201900186 PMC6548954 31179225

[15] K. Tao , Z. Chen , J. Yu , H. Zeng , J. Wu , Z. Wu , Q. Jia , P. Li , Y. Fu , H. Chang , W. Yuan , Adv. Sci. 2022, 9, e2104168. 10.1002/advs.202104168 PMC8981453 35098703

[16] C. Yang , H. Zhang , Y. Liu , Z. Yu , X. Wei , Y. Hu , Adv. Sci. 2018, 5, 1801070. 10.1002/advs.201801070 PMC6299731 30581706

[17] Z. Zhu , S.‐Z. Guo , T. Hirdler , C. Eide , X. Fan , J. Tolar , M. C. Mcalpine , Adv. Mater. 2018, 30, 1707495. 10.1002/adma.201707495 PMC6310159 29691902

[18] X. Chen , W. Jian , Z. Wang , J. Ai , Y. Kang , P. Sun , Z. Wang , Y. Ma , H. Wang , Y. Chen , X. Feng , Sci. Adv. 2023, 9, eadi0357. 37494444 10.1126/sciadv.adi0357 PMC10371014

[19] a) J. Zhao , R. Ghannam , K. O. Htet , Y. Liu , M.‐K. Law , V. A. L. Roy , B. Michel , M. A. Imran , H. Heidari , Adv. Healthcare Mater. 2020, 9, e2000779; 10.1002/adhm.202000779 32729228

[20] K. Veenuttranon , K. Kaewpradub , I. Jeerapan , Nanomicro Lett. 2023, 15, 85. 37002513 10.1007/s40820-023-01045-1 PMC10066049

[21] N. Ali , S. S. Hekal , Sol. Energy 2022, 3, 5.

[22] a) X. Yuan , R. Sun , Y. Wu , T. Wang , Y. Wang , W. Wang , Y. Yu , J. Guo , Q. Wu , J. Min , Adv. Funct. Mater. 2022, 32, 2200107;

[23] a) M. Silverå Ejneby , M. Jakesová , J. J. Ferrero , L. Migliaccio , I. Sahalianov , Z. Zhao , M. Berggren , D. Khodagholy , V. Derek , J. N. Gelinas , E. D. Glowacki , Nat. Biomed. Eng. 2022, 6, 741; 34916610 10.1038/s41551-021-00817-7

[24] a) S. Park , S. W. Heo , W. Lee , D. Inoue , Z. Jiang , K. Yu , H. Jinno , D. Hashizume , M. Sekino , T. Yokota , K. Fukuda , K. Tajima , T. Someya , Nature 2018, 561, 516; 30258137 10.1038/s41586-018-0536-x

[25] C. Han , J. Huang , A. Zhangji , X. Tong , K. Yu , K. Chen , X. Liu , Y. Yang , Y. Chen , W. Ali Memon , K. Amin , W. Gao , Z. Deng , K. Zhou , Y. Wang , X. Qi , Micromachines 2022, 13, 561. 35457866 10.3390/mi13040561 PMC9032666

[26] Y.‐S. Hsiao , Y.‐H. Liao , H.‐L. Chen , P. Chen , F.‐C. Chen , ACS Appl. Mater. Interfaces 2016, 8, 9275. 26999636 10.1021/acsami.6b00916

[27] S. Biswas , Y. Lee , H. Choi , H. Kim , JPhys Mater. 2023, 6, 2002.

[28] a) D. Seliktar , Science 2012, 336, 1124; 22654050 10.1126/science.1214804

[29] Y. S. Zhang , A. Khademhosseini , Science 2017, 356, eaaf3627. 28473537 10.1126/science.aaf3627 PMC5841082

[30] a) J. Zhu , H. Zhou , E. M. Gerhard , S. Zhang , F. I. Parra Rodriguez , T. Pan , H. Yang , Y. Lin , J. Yang , H. Cheng , Bioact. Mater. 2023, 19, 360; 35574051 10.1016/j.bioactmat.2022.04.020 PMC9062426

[31] T. Jiang , Q. Li , J. Qiu , J. Chen , S. Du , X. Xu , Z. Wu , X. Yang , Z. Chen , T. Chen , Int. J. Nanomed. 2022, 17, 3125. 10.2147/IJN.S372211 PMC9309282 35898438

[32] a) J. Yang , Y. Chen , L. Zhao , Z. Feng , K. Peng , A. Wei , Y. Wang , Z. Tong , B. Cheng , Composites, Part B 2020, 197, 108139;

[33] X. Chen , J. Chen , L. Huang , S. Nie , W. Xu , Y. Yin , S. Zhang , F. Pei , K. Yu , W. Su , Y. Wang , W. Yuan , Y. Li , Z. Cui , Adv. Mater. Technol. 2023, 8, 2201406.

[34] G. Wang , J. Zhang , C. Yang , Y. Wang , Y. Xing , M. A. Adil , Y. Yang , L. Tian , M. Su , W. Shang , K. Lu , Z. Shuai , Z. Wei , Adv. Mater. 2020, 32, 2005153. 10.1002/adma.202005153 33150635

[35] a) X. Yang , R. Sun , Y. Wang , M. Chen , X. Xia , X. Lu , G. Lu , J. Min , Adv. Mater. 2022, 35, 2209350; 10.1002/adma.202209350 36413076

[36] a) T. Zhang , C. An , Y. Xu , P. Bi , Z. Chen , J. Wang , N. Yang , Y. Yang , B. Xu , H. Yao , X. Hao , S. Zhang , J. Hou , Adv. Mater. 2022, 34, 2207009; 10.1002/adma.202207009 36070897

[37] L. Liu , Y. Yang , Y. Wang , M. A. Adil , Y. Zhao , J. Zhang , K. Chen , D. Deng , H. Zhang , K. Amin , Y. Wu , Y. Zhang , Z. Wei , ACS Mater. Lett. 2022, 4, 401.

[38] a) M. A. Adil , W. A. Memon , J. Zhang , M. J. Iqbal , C. Yang , Y. Wang , W. Zou , Z. Wei , Sol. RRL 2022, 6, 2101083;

[39] M. Rouabhia , H. J. Park , A. Abedin‐Do , Y. Douville , M. Méthot , Z. Zhang , J. Tissue Eng. Regener. Med. 2020, 14, 909. 10.1002/term.3040 32293799

[40] a) P. Chen , C. Xu , P. Wu , K. Liu , F. Chen , Y. Chen , H. Dai , Z. Luo , ACS Nano 2022, 16, 16513; 36174221 10.1021/acsnano.2c05818

[41] a) M. Verdes , K. Mace , L. Margetts , S. Cartmell , Curr. Opin. Biotechnol. 2022, 75, 102710; 35398709 10.1016/j.copbio.2022.102710

[42] R. Nuccitelli , P. Nuccitelli , C. Li , S. Narsing , D. M. Pariser , K. Lui , Wound Repair Regener. 2011, 19, 645. 10.1111/j.1524-475X.2011.00723.x PMC3228273 22092802

[43] M. Jakesová , T. A. Sjöström , V. Derek , D. Poxson , M. Berggren , E. D. Glowacki , D. T. Simon , npj Flexible Electron. 2019, 3, 14.

[44] W. Hu , X. Wei , L. Zhu , D. Yin , A. Wei , X. Bi , T. Liu , G. Zhou , Y. Qiang , X. Sun , Z. Wen , Y. Pan , Nano Energy 2019, 57, 600.

[45] J. Wang , P. Xue , Y. Jiang , Y. Huo , X. Zhan , Nat. Rev. Chem. 2022, 6, 614. 37117709 10.1038/s41570-022-00409-2

[46] Y. Cui , Y. Wang , J. Bergqvist , H. Yao , Y. Xu , B. Gao , C. Yang , S. Zhang , O. Inganäs , F. Gao , J. Hou , Nat. Energy 2019, 4, 768.

[47] a) X. Li , C. Yin , S. S. Liew , C.‐S. Lee , K. Pu , Adv. Funct. Mater. 2021, 31, 2106154;

[48] S. Li , S. Dong , W. Xu , S. Tu , L. Yan , C. Zhao , J. Ding , X. Chen , Adv. Sci. 2018, 5, 1700527. 10.1002/advs.201700527 PMC5980143 29876202

[49] A. Heyneman , H. Hoeksema , D. Vandekerckhove , A. Pirayesh , S. Monstrey , Burns 2016, 42, 1377. 27126813 10.1016/j.burns.2016.03.029

[50] a) C. Kalirajan , T. Palanisamy , Adv. Healthcare Mater. 2020, 9, e2000247; 10.1002/adhm.202000247 32378364

[51] S. Chernousova , M. Epple , Angew. Chem., Int. Ed. 2013, 52, 1636. 10.1002/anie.201205923 23255416

[52] F. Zhao , Y. Liu , T. Song , B. Zhang , D. Li , Y. Xiao , X. Zhang , J. Mater. Chem. B 2022, 10, 2135. 35262122 10.1039/d1tb02850b

[53] A. Ghavaminejad , C. H. Park , C. S. Kim , Biomacromolecules 2016, 17, 1213. 26891456 10.1021/acs.biomac.6b00039

[54] J. Jneid , N. Cassir , S. Schuldiner , N. Jourdan , A. Sotto , J.‐P. Lavigne , B. La Scola , Front. Cell Infect. Microbiol. 2018, 8, 282. 30155447 10.3389/fcimb.2018.00282 PMC6102383

[55] J. K. S. Tan , S. W. Song , J. Zeng , C. H. Lo , Bioeng. Transl. Med. 2022, 8, e10411. 36248233 10.1002/btm2.10411 PMC9538315

[56] J. Ouyang , X. Ji , X. Zhang , C. Feng , Z. Tang , N. Kong , A. Xie , J. Wang , X. Sui , L. Deng , Y. Liu , J. S. Kim , Y. Cao , W. Tao , Proc. Natl. Acad. Sci. USA 2020, 117, 28667. 33139557 10.1073/pnas.2016268117 PMC7682336

[57] P. Schilrreff , U. Alexiev , Int. J. Mol. Sci. 2022, 23, 4928. 35563319 10.3390/ijms23094928 PMC9104327

[58] A. S. Kimball , A. D. Joshi , A. E. Boniakowski , M. Schaller , J. Chung , R. Allen , J. Bermick , W. F. Carson , P. K. Henke , I. Maillard , S. L. Kunkel , K. A. Gallagher , Front. Immunol. 2017, 8, 635. 28620387 10.3389/fimmu.2017.00635 PMC5451506

[59] Y. Jin , R. H. Koh , S.‐H. Kim , K. M. Kim , G. K. Park , N. S. Hwang , Mater. Sci. Eng., C 2020, 115, 111096. 10.1016/j.msec.2020.111096 32600700

[60] A. Abedin‐Do , Z. Zhang , Y. Douville , M. Méthot , J. Bernatchez , M. Rouabhia , J. Tissue Eng. Regener. Med. 2022, 16, 643. 10.1002/term.3305 35442544

[61] L. Mao , S. Hu , Y. Gao , L. Wang , W. Zhao , L. Fu , H. Cheng , L. Xia , S. Xie , W. Ye , Z. Shi , G. Yang , Adv. Healthcare Mater. 2020, 9, e2000872. 10.1002/adhm.202000872 32864898

[62] a) M. S. Wietecha , L. A. Dipietro , Adv. Wound Care 2013, 2, 81; 10.1089/wound.2011.0348 PMC3623575 24527330

[63] S. Korntner , C. Lehner , R. Gehwolf , A. Wagner , M. Grütz , N. Kunkel , H. Tempfer , A. Traweger , Adv. Drug Delivery Rev. 2019, 146, 170. 10.1016/j.addr.2018.02.010 29501628

[64] J. M. Reinke , H. Sorg , Eur. Surg. Res. 2012, 49, 35. 22797712 10.1159/000339613

[65] D. G. Armstrong , E. B. Jude , J. Am. Podiatr. Med. Assoc. 2002, 92, 12. 11796794 10.7547/87507315-92-1-12

[66] M. Chang , T. T. Nguyen , Acc. Chem. Res. 2021, 54, 1080. 33596041 10.1021/acs.accounts.0c00864

[67] M. Gao , T. T. Nguyen , M. A. Suckow , W. R. Wolter , M. Gooyit , S. Mobashery , M. Chang , Proc. Natl. Acad. Sci. USA 2015, 112, 15226. 26598687 10.1073/pnas.1517847112 PMC4679041

[68] S. Li , Q. Fu , L. Meng , X. Wan , L. Ding , G. Lu , G. Lu , Z. Yao , C. Li , Y. Chen , Angew. Chem. 2022, 134, e202207397. 10.1002/anie.202207397 35765215

[69] J. Zhang , Y. Zhao , J. Fang , L. Yuan , B. Xia , G. Wang , Z. Wang , Y. Zhang , W. Ma , W. Yan , W. Su , Z. Wei , Small 2017, 13, 1700388. 10.1002/smll.201700388 28398016

[70] J. Xiang , L. Ma , Z. Gu , H. Jin , H. Zhai , J. Tong , T. Liang , J. Li , Q. Ren , Q. Liu , Cells 2022, 11, 1173. 35406737 10.3390/cells11071173 PMC8998031

[71] A. Nimmagadda , X. Liu , P. Teng , M. Su , Y. Li , Q. Qiao , N. K. Khadka , X. Sun , J. Pan , H. Xu , Q. Li , J. Cai , Biomacromolecules 2017, 18, 87. 28064500 10.1021/acs.biomac.6b01385 PMC5267617

[72] a) P. S. Babo , R. L. Pires , L. Santos , A. Franco , F. Rodrigues , I. Leonor , R. L. Reis , M. E. Gomes , ACS Biomater. Sci. Eng. 2017, 3, 1359; 33429694 10.1021/acsbiomaterials.6b00508

[73] Y. Liang , X. Zhao , T. Hu , B. Chen , Z. Yin , P. X. Ma , B. Guo , Small 2019, 15, e1900046. 30786150 10.1002/smll.201900046

[74] Z.‐X. Zhou , W. Hu , Z. Zhao , H. Fu , ACS Appl. Mater. Interfaces 2022, 14, 46313. 36194167 10.1021/acsami.2c13592

[75] R. Ansari , A. F. Delavar , J. Appl. Polym. Sci. 2009, 113, 2293.

[76] M. C. Deeds , J. M. Anderson , A. S. Armstrong , D. A. Gastineau , H. J. Hiddinga , A. Jahangir , N. L. Eberhardt , Y. C. Kudva , Lab. Anim. 2011, 45, 131. 21478271 10.1258/la.2010.010090 PMC3917305

[77] X. Li , F. He , X. Tuo , Y. Qiu , J. Guo , Y. Wu , X. Meng , Z. Yang , Front. Cell Infect. Microbiol. 2022, 12, 935681. 36061878 10.3389/fcimb.2022.935681 PMC9437313

[78] F. Zhu , H. Zheng , S. Chen , K. Zhang , X. Qin , J. Zhang , T. Liu , Y. Fan , L. Wang , X. Li , J. Zhang , W. Xu , Nat. Commun. 2022, 13, 3208. 35680915 10.1038/s41467-022-30988-z PMC9184642

[79] B. N. Akerberg , F. Gu , N. J. Vandusen , X. Zhang , R. Dong , K. Li , B. Zhang , B. Zhou , I. Sethi , Q. Ma , L. Wasson , T. Wen , J. Liu , K. Dong , F. L. Conlon , J. Zhou , G.‐C. Yuan , P. Zhou , W. T. Pu , Nat. Commun. 2019, 10, 4907. 31659164 10.1038/s41467-019-12812-3 PMC6817842

[80] W. Gao , J. Yang , R. Liu , Y. Yan , C. Xie , J. Yu , K. Tang , Rapid Commun. Mass Spectrom. 2020, 34, e8817. 32335952 10.1002/rcm.8817

[81] a) J. Ma , T. Chen , S. Wu , C. Yang , M. Bai , K. Shu , K. Li , G. Zhang , Z. Jin , F. He , H. Hermjakob , Y. Zhu , Nucleic Acids Res. 2019, 47, D1211; 30252093 10.1093/nar/gky869 PMC6323926

[82] a) W. Zeng , S. Zheng , Y. Mao , S. Wang , Y. Zhong , W. Cao , T. Su , M. Gong , J. Cheng , Y. Zhang , H. Yang , Front. Pharmacol. 2021, 12, 805499; 35002739 10.3389/fphar.2021.805499 PMC8728018

[83] W. Deng , Z. Su , P. Liang , Y. Ma , Y. Liu , K. Zhang , Y. Zhang , T. Liang , J. Shao , X. Liu , W. Han , R. Li , Emerging Microbes Infect. 2021, 10, 1272. 10.1080/22221751.2021.1942228 PMC8238073 34120578

[84] S. Yang , H. Li , H. Yao , Y. Zhang , H. Bao , L. Wu , C. Zhang , M. Li , L. Feng , J. Zhang , Z. Zheng , G. Xu , F. Wang , Cell Death Differ. 2021, 28, 2351. 33664479 10.1038/s41418-021-00756-5 PMC8329214

