# Supplementary material for: Flexible Organic Photovoltaic‐Powered Hydrogel Bioelectronic Dressing With Biomimetic Electrical Stimulation for Healing Infected Diabetic Wounds
Source: Adv Sci (Weinh). 2023 Dec 25;11(10):2307746. doi: 10.1002/advs.202307746 (PMC10933690; doi:10.1002/advs.202307746)
Supplement: Supplementary file 1 — Supporting Information [file ADVS-11-2307746-s001.pdf]

ADVANCED SCIENCE
Open Access

Supporting Information

for *Adv. Sci.*, DOI 10.1002/advs.202307746

Flexible Organic Photovoltaic-Powered Hydrogel Bioelectronic Dressing With Biomimetic Electrical Stimulation for Healing Infected Diabetic Wounds

Yi-Wei Hu, Yu-Heng Wang, Fang Yang, Ding-Xin Liu, Guang-Hao Lu, Sheng-Tao Li, Zhi-Xiang Wei, Xiang Shen, Zhuang-De Jiang, Yi-Fan Zhao, Qian Pang, Bai-Yang Song, Ze-Wen Shi, Shareen Shafique, Kun Zhou, Xiao-Lian Chen, Wen-Ming Su, Jia-Wen Jian, Ke-Qi Tang, Tie-Long Liu and Ya-Bin Zhu**

Supporting Information

Flexible Organic Photovoltaic-Powered Hydrogel Bioelectronic Dressing with Biomimetic Electrical Stimulation for Healing Infected Diabetic Wounds

Yi-Wei Hu, Yu-Heng Wang, Fang Yang, Ding-Xin Liu, Guang-Hao Lu, Sheng-Tao Li, Zhi-Xiang Wei, Xiang Shen, Zhuang-De Jiang, Yi-Fan Zhao, Qian Pang, Bai-Yang Song, Ze-Wen Shi, Shareen Shafique, Kun Zhou, Xiao-Lian Chen, Wen-Ming Su, Jia-Wen Jian, Ke-Qi Tang, Tie-Long Liu, Ya-Bin Zhu**

Characterization of the Flexible Organic Photovoltaic (OPV) Cells

In this work, the space charge limited current (SCLC) methodology has been employed to evaluate the electron (μ e) and hole (μ h) carrier mobility of all the concerned devices. Hence, for the systems mentioned above, **Figure S1**d,e exhibit the corresponding curves whereas Table S1 demonstrates the relevant mobility details. Consequently, an increase in the μ e and μ h values of the binary devices has been observed. Hence, μ h and μ e values from $1.43E^{-3}$ to $5.30E^{-3}$ and $2.04E^{-4}$ to $5.26E^{-4}$ $cm^2\ V^{-1}\ s^{-1}$, respectively, have been attained for the corresponding devices. Similarly, the TPE molecule has successfully imparted its excellent mobility attributes to the parent PM6 system as a significant enhancement in both the μ h and μ e values in the ternary blends has been observed.

Likewise, the recombination mechanisms have also been evaluated for the corresponding devices as such processes play a great role in determining the final fill factor (FF) values. Thus, both binary and ternary devices with varied polymer molecular weights have been analyzed by determining the light intensity dependence upon the J–V characteristics. A power law, $J_{sc} \propto I_{\alpha}$, is generally used to determine the relationship between the light illumination intensity and the J_{sc} (the short-circuit current), where α close to unity represents extremely frail bimolecular recombination that can be ignored. Thus, by plotting a log–log graph between the light intensity and the J_{sc} for the corresponding binary and ternary devices, fabricated by utilizing PM6 with varied molecular weights (Figure S1g), it can be seen that all systems exhibit an α value close to unity, indicating weak bimolecular recombination. A closer look reveals that for both binary and ternary systems, the increment in TPE leads to a little bit of lower degree of bimolecular recombination in the corresponding system. Accordingly, by comparing the slopes obtained for the binary and ternary devices, the latter systems demonstrate the reduced recombination.

Moreover, to acquire further insight, the charge collection and exciton dissociation efficiency for all the involved devices have been evaluated through plotting an effective voltage (V_{eff}) versus photocurrent density (J_{ph}) graph (Figure S1f), where the J_{ph} is defined as $J_{ph} = J_L - J_D$ (J_L and J_D are the current density under light illumination and in the dark, respectively) and the

V_{eff} is defined as $V_{eff} = V_0 – V$ (V is the applied bias and V_0 is the voltage at which $J_{ph} = 0$). Ultimately, the charge collection and exciton dissociation efficiency can be estimated by using the saturation current density (J_{sat}) as J_{ph}/J_{sat}. Hence, from the curve, the J_{ph}/J_{sat} values of the binary devices turn out to be 98.03%. Likewise, the ternary devices exhibit enhanced charge collection and exciton dissociation efficiencies as J_{ph}/J_{sat} values of 98.94% have been attained. Hence, devices with higher Mw revealed better exciton dissociation and charge transport characteristics for both binary and ternary systems. Likewise, a comparison based on active layer components reveals a better exciton dissociation and charge collection in the ternary blends, which might explain the higher J_{sc} values in the concerning devices.

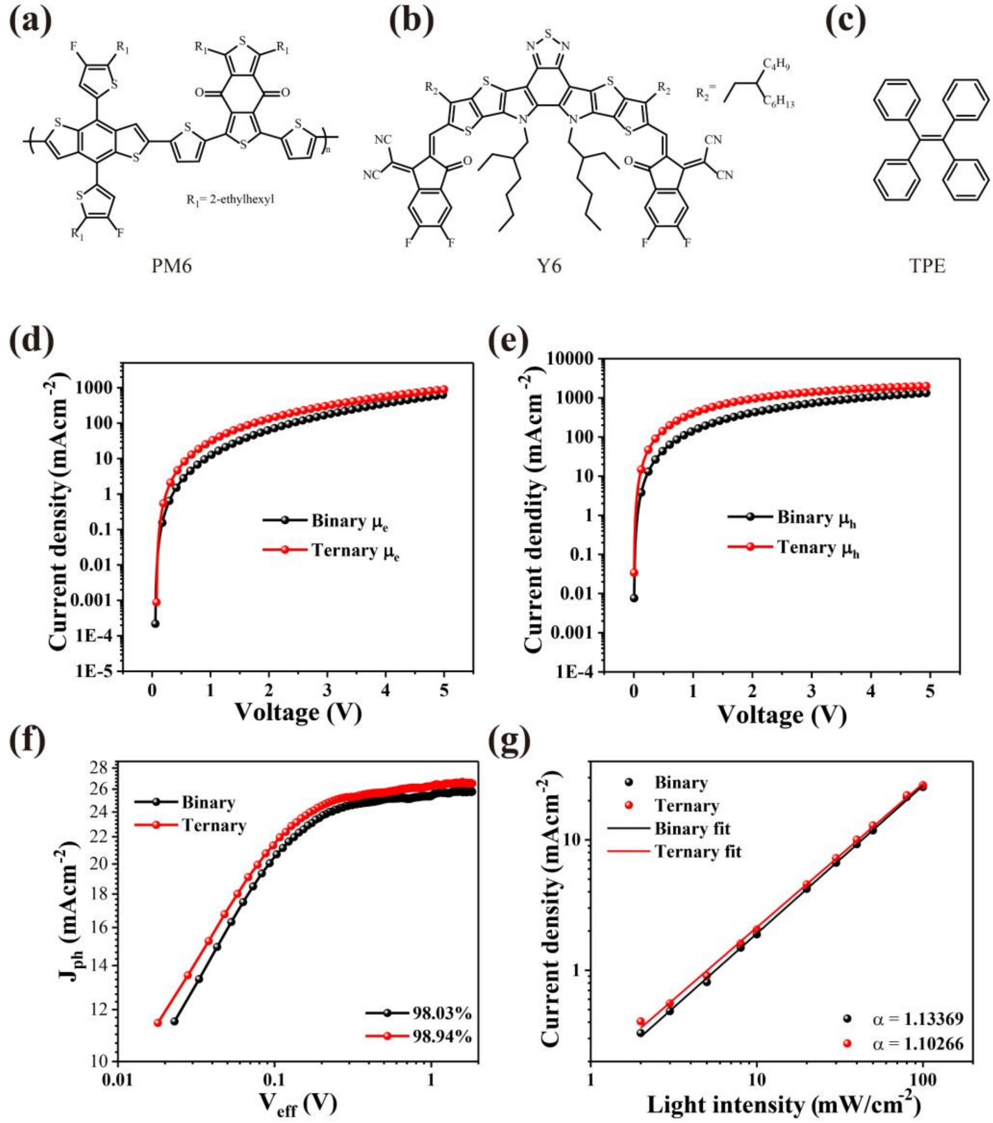

Figure S1. Molecular formulas of (a) PM6, (b) Y6 and (c) TPE. (d, e) Electron mobility and hole mobility. (f) Photocurrent density versus effective voltage curves. (g) Photocurrent density at $V_{eff} = 0.1$ V versus light intensity for the ternary systems.

Table S1. Electron mobility and hole mobility of the flexible OPV cells.

**Device**	**μe**	**μh**
Binary	$2.04E^{-4}$	$1.43E^{-3}$
Ternary	$5.26E^{-4}$	$5.30E^{-3}$

Table S2. The summary of reported electrical stimulation therapies for wound healing.

Intensity of ES	Duration of ES	Type of ES	Wound model	Direction of ES	Electrode property	Ref.
300 mV mm^{-1}, 2 Hz pulse frequency	15 min/ day (one time per day) for 5 days	Pulsed current (PC)	Acute wound in rat	Laterally across the wound	Silicon rubber/carbon electrodes	[1]
2000 mV mm^{-1}, 5 Hz pulse frequency	1 min/ day (two times per day) for 5 days	PC	Thermal wound injury in rat	Laterally across the wound	Silicon rubber/carbon electrodes	[2]
100 mV mm^{-1}, continuous	Continuous application for 7/11/14 days	Direct current (DC)	Acute wound in Bama miniature pig	ES from the periphery to the center	Hollow carbon fibre ring and needle-shaped flexible electrodes	[3]
100 mV mm^{-1}, continuous	Continuous application for 7/14 days	DC	Acute wound in Bama miniature pig	ES from the periphery to the center	Polyurethane membrane containing silver nanowires	[4]
150 mV mm^{-1}, continuous	60 min/ day (one time per day) for 5 days	DC	Acute wound in rat	Laterally across the wound	Bacterial cellulose/gelatin membrane	[5]
100 mV mm^{-1}, continuous	60 min/ day (one time every two days) for 14 days	DC	Acute wound in rat	Laterally across the wound	Bacterial cellulose/MXene hydrogel	[6]
70-90 mV mm^{-1}, continuous	12 h/ day for 12 days	DC	Acute wound in mouse	ES from the periphery to the center	Hollow metal ring electrode	[7]

Table S3. The summary of reported power supply devices for biomedical applications.

Device	Power supply principles	Output intensity | duration of the power supply device	Application	Ref.
FPSC [a)]	Photovoltaic (perovskite solar cell)	215 μW cm^{-2} (under 600lux LED illumination) | Continuous	Powering autonomous wearable biosensors	*Nat Electron*[8]
OPV cells [b)]	Photovoltaic (OPV cells)	100 μA cm^{-2} (under 626 nm LED array with diffusor, single OPV cell) | Continuous	Power the organic electronic ion pump	*npj Flex Electron*[9]
P3HT NPs [c)]	OPV cells	20 mV (under 540nm light stimulation, 40 mW mm^{-2}) | Continuous	Forming light-sensitive interfaces with retinal neurons to rescue visual acuity of rats with retinitis pigmentosa	*Nat Nanotechnol*[10]
iTENG [d)]	Triboelectric effect	2 V | 24 h	Promoting the migration of fibroblast (L929)	*Nano Energy*[11]
RD-TENG [e)]	Triboelectric effect	160 V | 6000 pulses/day for 6 days	Promoting the proliferation and migration of fibroblast (L929)	*Nano Energy*[12]
TENG	Triboelectric effect	4.7 V | 6 days	Forming a part of electrogenerative dressing to promote healing of pig deep skin wounds	*Adv Mater*[13]
iBEDs [f)]	Piezoelectric effect	6.5 V (under 26.2% bending strain) | Continuous	Powering tissue-adhesive piezoelectric soft sensor	*Adv Funct Materials*[14]
HPSP [g)]	Piezoelectric effect	0.5 V | 6 days	Enhancing the healing of acute skin lesions in mice, promoting the growth and migration of fibroblast (NIH/3T3)	*Nano Research*[15]
Sm-PUEH [h)]	Ultrasound-driven piezoelectric effect	2 V | 5 min	Deep brain stimulation and periaqueductal gray activation in rats	*Sci Adv*[16]
PLA/KNN @PDA nanofibers [i)]	Ultrasound-driven piezoelectric effect	17.9 V (under a pressure of 0.5 N, 10 Hz) | Continuous	Repairing spinal cord injury in rats	*ACS Nano*[17]
Button cell	Dry battery	40 V | 1 h per time	Promoting the wound healing of type 2 diabetic rats	*Bioact Mater*[18]
Wireless bioresorbable stimulator	Radio frequency (RF) energy	15.3 mV | 1 h	Promoting the healing of transected tibial nerve in rats	*Adv Funct Mater*[19]
IBMN [j)]	RF energy	1 V | 1h per day for 9 days	Promoting the damage repair of rat skeletal muscle, promoting the migration of myoblasts (C2C12)	*Nano Lett*[20]

a) FPSC, flexible perovskite solar cell; b) OPV, organic photovoltaic; c) P3HT NPs, poly[3-hexylthiophene] nano particles; d) iTENG, implantable triboelectric nanogenerators; e) RD-TENG, rotatory disc-shaped triboelectric nanogenerators; f) iBEDs, implantable bioelectric devices; g) HPSP, hybrid patch with self-adhesive and piezoelectric nanogenerator; h) Sm-PUEH, Sm-PMN-PT single crystal–based piezoelectric US energy-harvesting; i) PLA/KNN@PDA, poly(lactic acid)/$K_{0.5}Na_{0.5}NbO_3$@polydopamine; j) IBMN, implantable and bioresorbable microneedle.

Characterization of the Flexible MEMS Electrode

The circuits fabricated on the substrate are composed of two parts, an outer high-conductivity region and an inner microcircuit guiding network (**Figure S2**a). The microelectronic guiding network is hexagonal in shape, with a width of approximately 3.5 μm for the circuit. The outer high-conductivity region connects to the anode of the OPV cells, while the inner microcircuit connects to the tissue to serve as the cathode, guiding the current to flow from the surrounding high conductive area to the central region, thus enabling the simulation of physiological damage currents. The results of the stress finite element analysis for MEMS electrodes and PMH dressing show that under deformation conditions, most of the stress is concentrated around the circuit with minimal impact on organic photovoltaic cells and MEMS electrodes (Figure S2b-d).

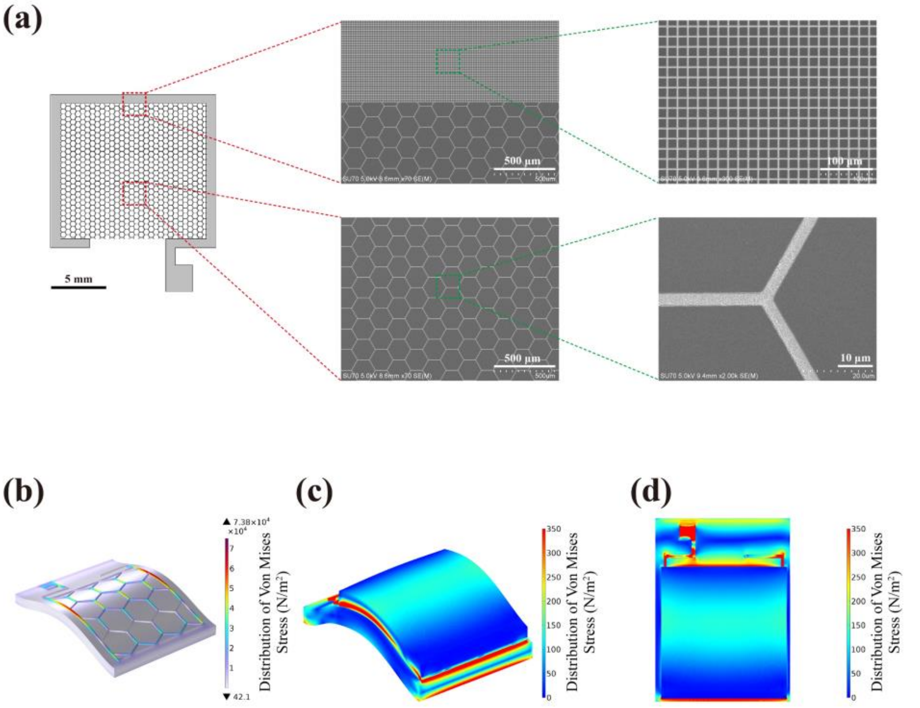

Figure S2. (a) Observation of the flexible MEMS electrode using SEM, focusing on the structure of central hexagonal grid-like microcircuit and surrounding high-conductivity regions. (b-d) Stress finite element analysis of the flexible MEMS electrode and the sandwich-structured PMH dressing.

Biocompatibility Assessment of the Flexible OPV Cell and MEMS Electrode

We have assessed the biocompatibility of the flexible OPV cell and MEMS microcircuit electrodes. **Figure S3**a demonstrates that the MEMS electrodes exhibit no toxicity towards human keratinocyte cells (HaCaT). These cells are able to adhere to and proliferate on the electrode surface. In co-culture experiments involving photovoltaic cells or MEMS electrodes, we observed no impact on HaCaT cell proliferation. Live/Dead staining show that HaCaT cells

maintain a high viability (>95%) when cultured on photovoltaic cells and MEMS electrodes, with no significant difference compared to cells grown on standard cell culture plates (TCP) (Figure S3b,f). Additionally, the hemolysis experiments (Figure S3c-d) demonstrate that the extract media from photovoltaic cells and MEMS electrodes do not have any substantial hemolytic effect on rat blood cells (hemolysis rate <5%). These results suggest that these devices exhibit no toxicity and appear to be biocompatible.

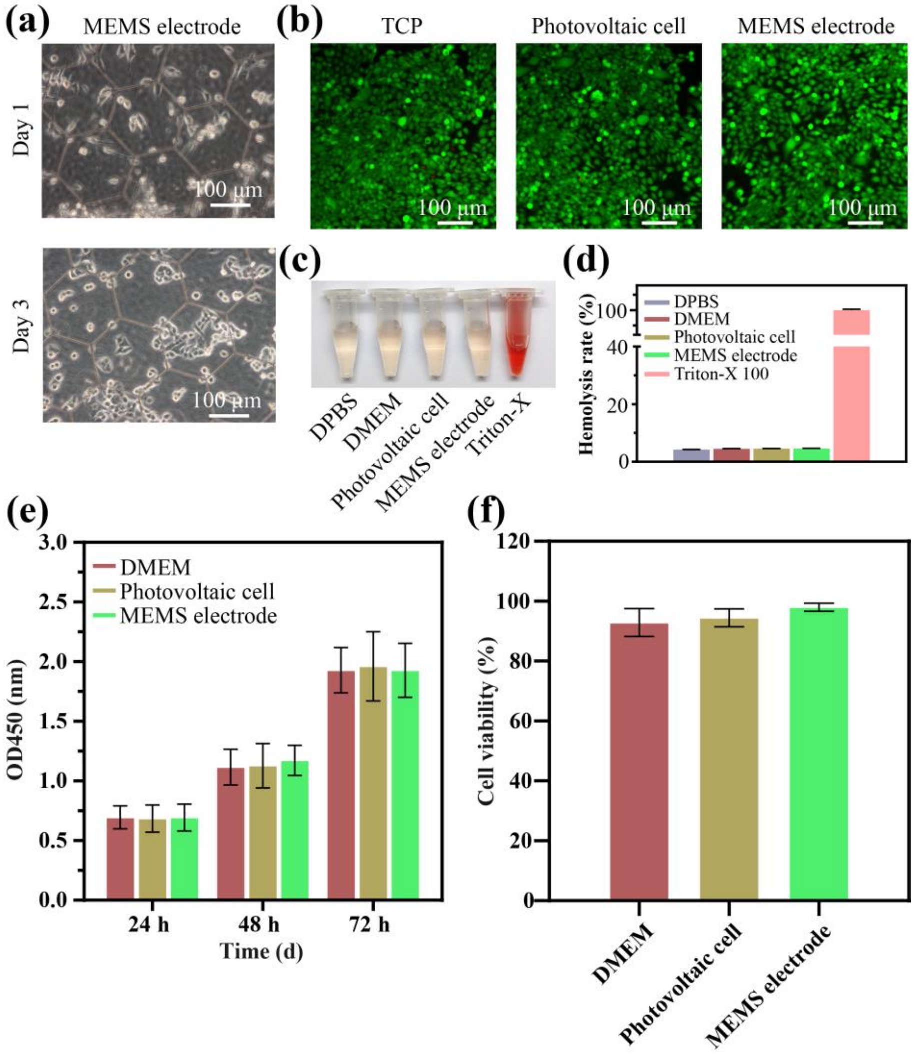

Figure S3. (a) The morphology of HaCaT cells adhering to and growing on the MEMS electrode surface after 1 and 3 days. (b) The Live/Dead staining results of cells co-cultured with flexible photovoltaic cells or MEMS electrodes for 72 h. (c, d) The results of hemolysis experiments. (e) Cell proliferation when co-culture with the devices for 24, 48 and 72 h. (f) Cell viability of cells co-cultured with flexible photovoltaic cells or MEMS electrodes for 72 h. The data were presented as mean ± SD (n=3).

Preparation of HAMA/DA-Ag (HD-Ag) Hydrogel

A variety of multifunctional hydrogels have been reported for wound healing applications, typically consisting of a hydrogel matrix combined with functional components. The overall strategy of the hydrogel preparation is presented in **Figure S4**a. Due to its promising cytocompatibility and inherent bioactivity, hyaluronic acid (HA) has been widely used in tissue engineering. Of HA derivatives, HAMA stands out as the most studied one[21]. Under certain photo-initiator and UV-light irradiation conditions, it is able to polymerize into a hydrogel. HAMA was synthesized by esterification of the primary alcohol group with methacrylic anhydride as reported elsewhere[22]. DA was then grafted onto the polymer chains of HAMA using 1-(3-dimethylaminopropyl)-3-ethylcarbodiimide hydrochloride (EDC)/N-hydroxysuccinimide (NHS) chemistry to ensure good integration between MEMS electrode patch and hydrogel[23]. This conjugation was further verified by ^{1}H NMR analysis (Figure S4b), which confirmed the successful methacrylation of HA and conjugation of dopamine to HAMA (HAMA-DA). Exhibiting excellent electrical conductivity[24] (up to 6.3×10^{7} S m^{-1}) with low toxicity to mammalian cells and a wide range of antibacterial activity, silver nanoparticles (AgNPs) are an ideal nanomaterial as conductive fillers of conductive hydrogel. The AgNPs were synthesized rapidly *in situ* by reducing silver ions through the reduction reaction induced by catechol groups [25] and UV light exposure [26]. At the same time, the HAMA-DA hydrogel is formed.

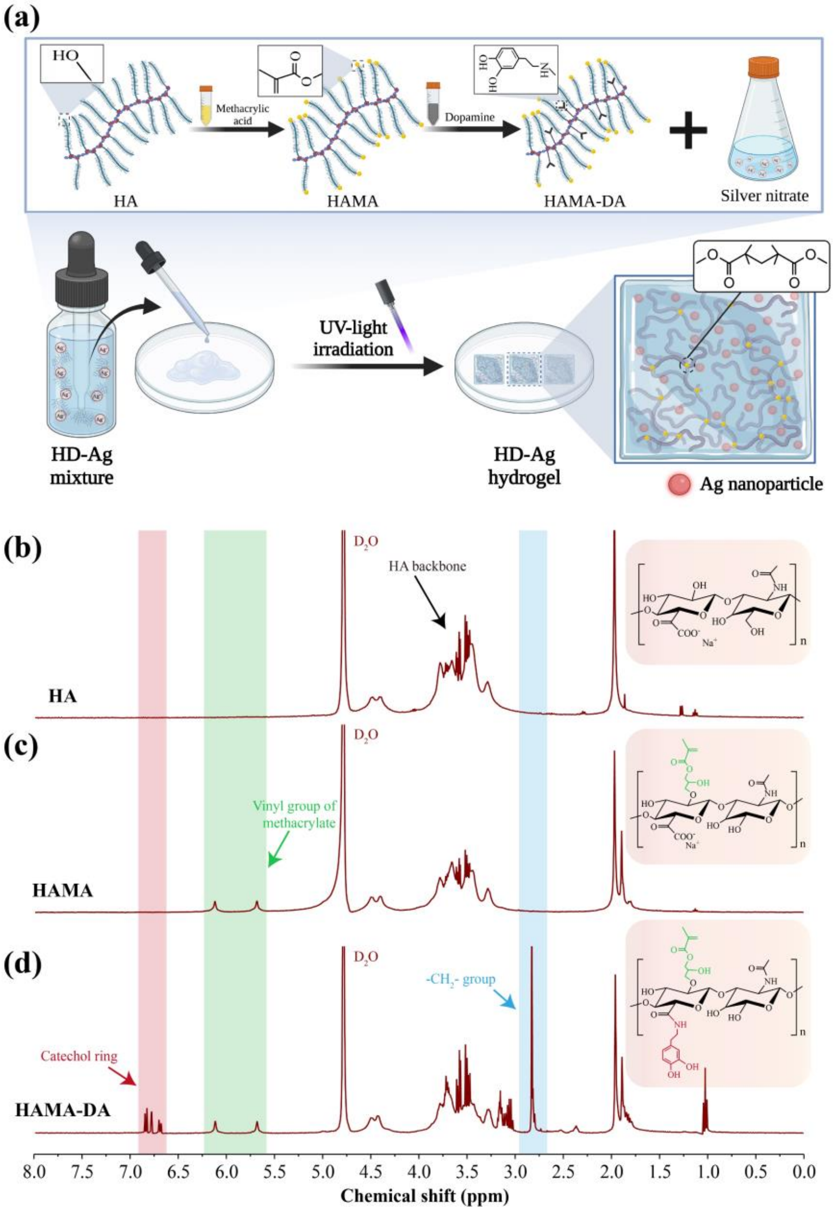

Figure S4. Schematic of preparation of HD-Ag hydrogel (a) and the ^{1}H NMR results of (b) HA, (c) HAMA and (d) HAMA-DA. (a) HAMA was synthesized by methacrylic anhydride esterification under alkaline environment. Dopamine modified HAMA (HAMA-DA, HD) was prepared by the EDC-NHS chemistry. Antibacterial and conductive HD-Ag hydrogel was obtained by photo-crosslinking induced by UV irradiation, where AgNPs were formed *in situ* by dopamine- and UV-mediated reduction reaction. (b, c, d) Samples were dissolved in deuterated water (D_2O) at a concentration of 1% (w/v). Peaks at δ5.7-6.1 ppm (marked with green) indicated the vinyl groups introduced by methacrylation (b-c). The protons in the catechol ring newly appeared at δ6.6-6.9 ppm (marked with red), and the peak at δ2.82 ppm (marked with blue) showed a $-CH_2-$ group closed to the catechol ring (d).

Characterization of HD-Ag Hydrogel

The HD-Ag hydrogels was examined via SEM and TEM. As **Figure S5**a reveals, the surface of the hydrogel exhibits a rough texture, containing numerous pores. No particles were observed in the pure HD hydrogel, whereas AgNPs were noted to be present in the HD-Ag hydrogels (Figure S5b-d), with an increase proportionally correlated to the concentration of AgNPs. The presence of AgNPs in the hydrogel system was depicted through TEM observation (Figure S5e). It was apparent that AgNPs show a widespread distribution in the hydrogel and there is no occurrence of aggregation. The average size of AgNPs found in HD-Ag_1, HD-Ag_2 and HD-Ag_3 samples was 44.20 ± 13.02, 48.00 ± 10.32 and 38.44 ± 9.43 nm, respectively.

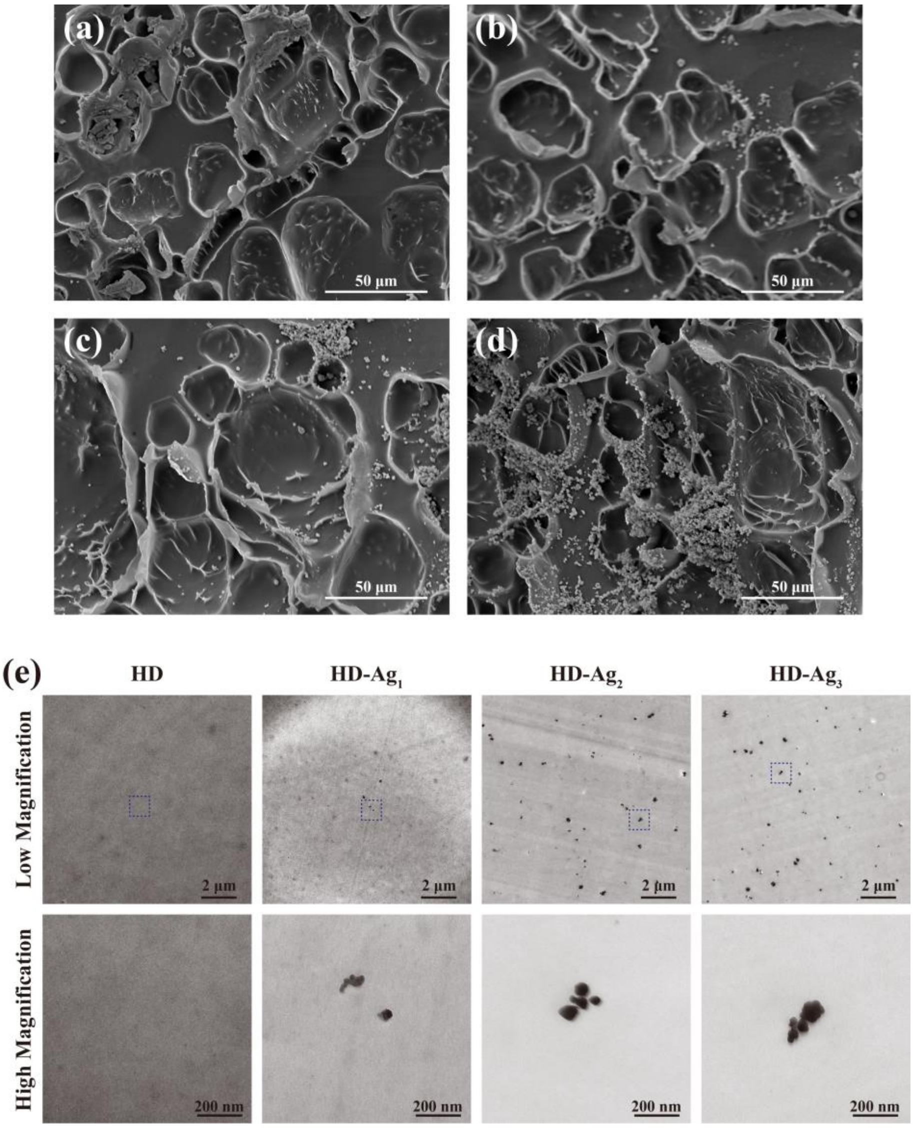

Figure S5. Surface morphology of the (a) HD, (b) HD-Ag_1, (c) HD-Ag_2 and (d) HD-Ag_3 hydrogels observed by SEM. (e) TEM images showing Ag nanoparticles are evenly dispersed in hydrogel matrix.

Figure 3a depicts the release profile of Ag^+ from HD-Ag hydrogels *in vitro*. During the initial 24 h, silver ions were released rapidly; afterwards, the ion discharge slowed and concentrations reached stable levels at 1.47 ppm (HD-Ag_1), 2.96 ppm (HD-Ag_2) and 4.24 ppm (HD-Ag_3) after 72 h. Figure 3b highlights the swelling behavior of these HD-Ag hydrogels, with each reaching their peak water absorption (~300% initial weight) after 96 h. Specifically, the highest swelling ratio was 318.1% in HD, followed by 314.6%, 307.3% and 299.3% in HD-Ag_1, HD-Ag_2 and HD-Ag_3, respectively. Even though there seems to be a minor decrease in the swelling rate with an increase in silver content, the differences among the various groups are minimal, all exhibiting excellent swelling capabilities. Recent research indicates that the toxicity of AgNPs is closely associated with the size and concentration of the nanoparticles. Smaller AgNPs exhibits stronger toxicity. Gliga et al. discovered that AgNPs with a 10 nm diameter exhibits significant cytotoxicity, while AgNPs around 50 nm in diameter is mildly toxic.[27] Another research demonstrated that at a concentration of AgNPs of 10 μg mL^{-1}, the cell viability of HaCaT was 98.76%, but the toxicity significantly increased when the concentration rose to 100 ug mL^{-1}. [28] In our research, the AgNPs synthesized in situ in the HD-Ag hydrogel were approximately 30-50 nm in diameter, thus less toxic than the smaller-sized (10 nm diameter) AgNPs.[29] Furthermore, AgNPs synthesized were mostly confined within the hydrogel system, limiting direct contact with cells. On the other hand, the silver accumulated in the organs such as liver and spleen can mostly be cleared within a few days. Roughly 70.5-98.6% of administered AgNPs were excreted in feces following oral administration, and under 0.5% of the administered dose was detected in the liver, spleen, intestines, or urine at 48 hours.[30] Therefore, no toxicity or significant tissue accumulation of AgNPs was observed in the treated mice. Considering the severe consequences of non-healing diabetic wounds, the minor toxicity associated with silver treatment can be considered tolerable.

X-ray photoelectron spectroscopy (XPS) analysis of a sample of HD-Ag_2 with a moderate concentration of Ag was performed to determine the valence state of silver within the hydrogel. The Ag 3d XPS spectra for HD-Ag_2 were fitted to two superimposed doublets (**Figure S6**). The peaks at 368.62 and 374.66 eV signified elemental Ag, whereas those observed at 362.22 and 374.26 eV represented Ag^+ ions, which confirmed the presence of both AgNPs and Ag^+ ions in the hydrogel. As shown in **Figure S7**, the rheological properties of HD and HD-Ag hydrogels cured via UV light were measured. When exposed to UV light, HD and HD-Ag solutions exhibited immediate gel-like properties (G'> G") with both moduli increasing over time. A sol-gel gelation process was observed with G'-G" crossover point (i.e., the gel point) within 1 second, and the photopolymerization reactions finished on reaching plateau modulus after 20

seconds. This result demonstrates good photosensitivity and photopolymerization ability of the HD and HD-Ag hydrogels, making them suitable for bioelectronic wound dressing applications.

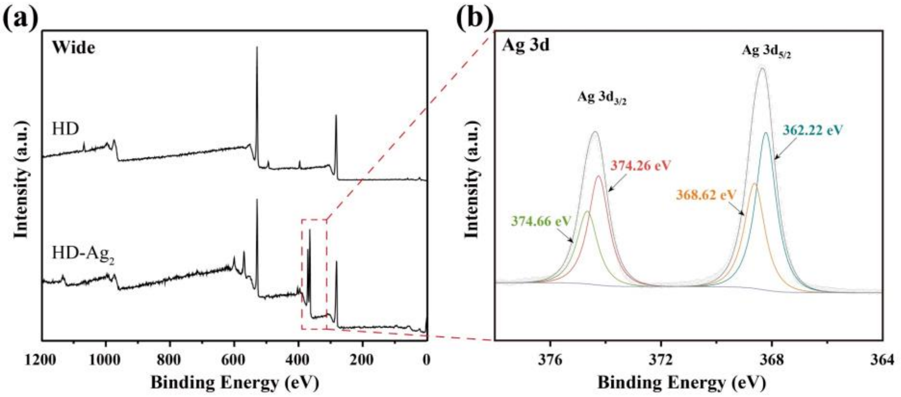

Figure S6. (a) XPS wide-scan spectra of HD and HD-Ag$_2$ hydrogel. (b) Ag 3d spectra of HD-Ag$_2$ hydrogel.

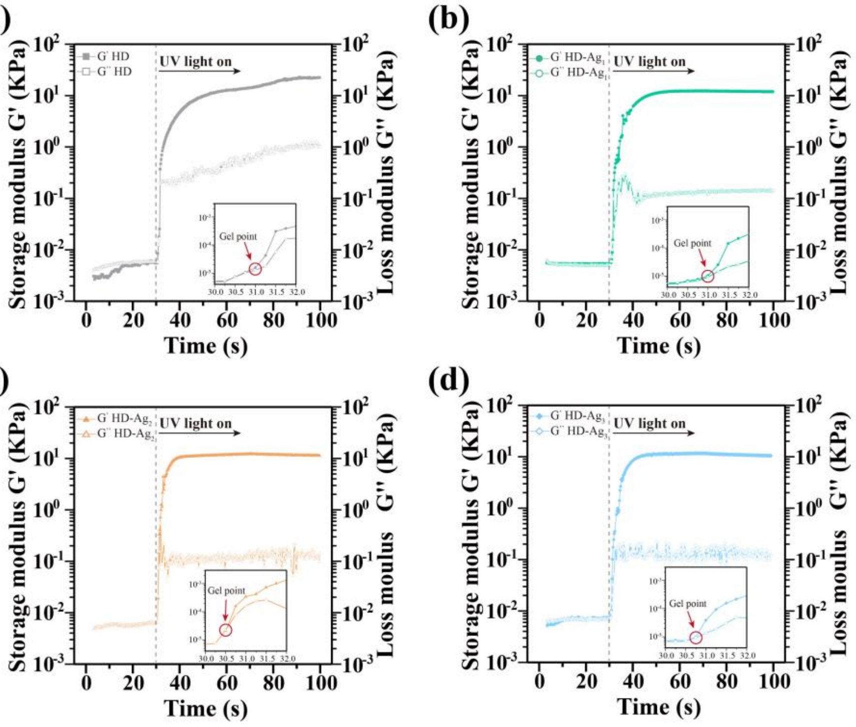

Figure S7. Rheological properties of (a) HD, (b) HD-Ag$_1$, (c) HD-Ag$_2$ and (d) HD-Ag$_3$ hydrogels as a function of time (time sweep) with UV curing.

The conductivities of the HD-Ag hydrogels were tested using an electrochemical workstation. As depicted in Figure 3c, the pure HD hydrogel had a relatively low conductivity (~0.04 S m^{-1}), mainly due to the weak ionic conductivity of hyaluronic acid. After incorporation of AgNPs, however, there was a dramatic increase in conductivity values from 0.07 to 0.27 and

0.43 S m^{-1} for HD-Ag_1, HD-Ag_2 and HD-Ag_3, respectively, owing to the establishment of an electronic conducting path by AgNPs. The enhanced electrical conducing abilities, particularly of the latter two formulations, were superior to many other materials reported in literatures [31]; hence making them ideal candidates for creating seamless interfaces between biology and electronics. The conductivity of HD-Ag hydrogels was further tested in a light-emitting diode (LED) contained circuit (**Figure S8**). Four parallel circuits were set up in this experiment, each incorporating an LED bulb and a hydrogel sample (Figure S8a). The bulbs remained off when the external DC power supply was turned off (Figure S8b). Upon activation of the power, all LEDs illuminated albeit with varying intensities among them (Figure S8c), which became even more apparent in dimly lit conditions. This result suggests that conductivity of HD-Ag hydrogels is tunable and increases with the increase of AgNPs content.

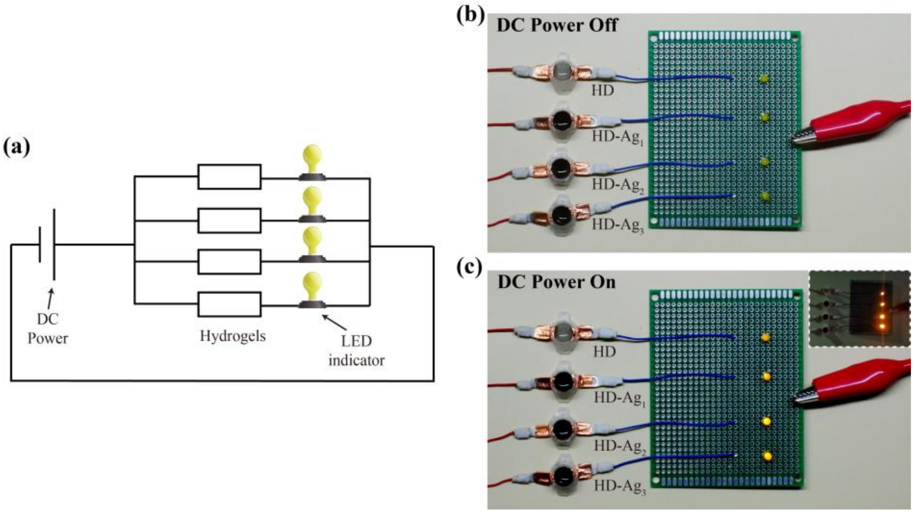

Figure S8. (a) Circuit diagram of the hydrogel conductivity test. (b, c) The hydrogel-integrated parallel circuit lighted up the LEDs after power up. The insert in (c) shows the brightness of the LEDs in a dark environment.

Biocompatibility of HD-Ag Hydrogel

To evaluate the biocompatibility of HD-Ag hydrogels, fibroblasts were adopted to be one cell model, because fibroblasts are known as an essential source for extracellular matrix (ECM) and play a key role in both wound healing and tissue remodeling. On the other hand, epithelial plays an important part in wound closure. Therefore, in this work, we selected basal layer keratinocytes (HaCaTs) and dermal fibroblasts (NIH/3T3) to evaluate biocompatibility of HD-Ag hydrogels via live/dead staining and Cell counting kit-8 (CCK-8) assay. Figure 3d revealed that all cells co-cultured with HD, HD-Ag_1 and HD-Ag_2 hydrogels for 3 days remained alive, though there were few dead cells. Conversely, there were visible signs of irregularly shaped live cells as well as many dead ones in HD-Ag_3 sample. The quantitative analysis demonstrated

a marked decrease in green fluorescence intensity and a corresponding rise in red fluorescence intensity for cells co-cultured with HD-Ag_3 (**Figure S9**). The cell proliferation assay (CCK-8) revealed an inhibitory effect of HD-Ag hydrogels (HD-Ag_1 and HD-Ag_2) on NIH/3T3 cell growth at the early stage (day 1), but this inhibitory effect weakened at days 3 and 5 (Figure 3g). No notable inhibitory effect was observed when keratinocytes were cultured with HD, HD-Ag_1 and HD-Ag_2 hydrogel throughout the entire culture period. In contrast, significant cytotoxicity of the HD-Ag_3 hydrogel towards both NIH/3T3 and keratinocytes was observed (Figure 3g-h). These results demonstrate good cytocompatibility of HD, HD-Ag_1 and HD-Ag_2 hydrogels to both fibroblasts and keratinocytes whereas significant toxicity is associated with HD-Ag_3 hydrogel. The biocompatibility in vivo was evaluated via subcutaneous implantation assay (**Figure S10**). Histological examination through H&E staining revealed differential degradation of the four hydrogel types following 7-day implantation. It was remarked that complete degradation had occurred within 7 days in HD, HD-Ag_1 and HD-Ag_2 group, and few granulocytes could be observed near their respective sites. Conversely, partial disintegration of hydrogel alongside a large number of local-region granulocytes infiltrated into surrounding tissue was observed in HD-Ag_3 group, indicating a severe local inflammatory response caused by the implantation of this material.

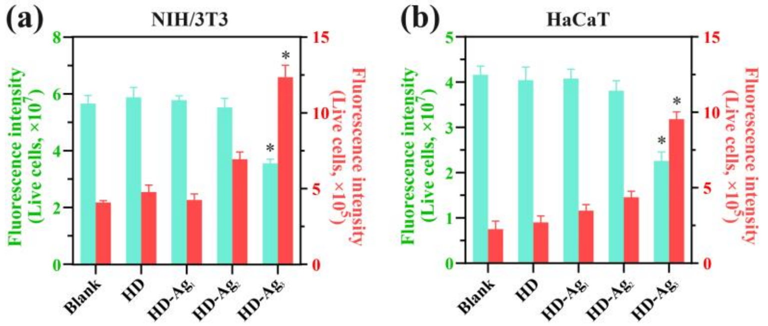

Figure S9. Quantitative comparison of the fluorescence intensity of (a) NIH/3T3 cells and (b) HaCaT cells by Live/Dead staining assay. Data were represented as mean ± SD, and error bar indicates SD (n=3, $*p<0.05$ when compared with Blank group).

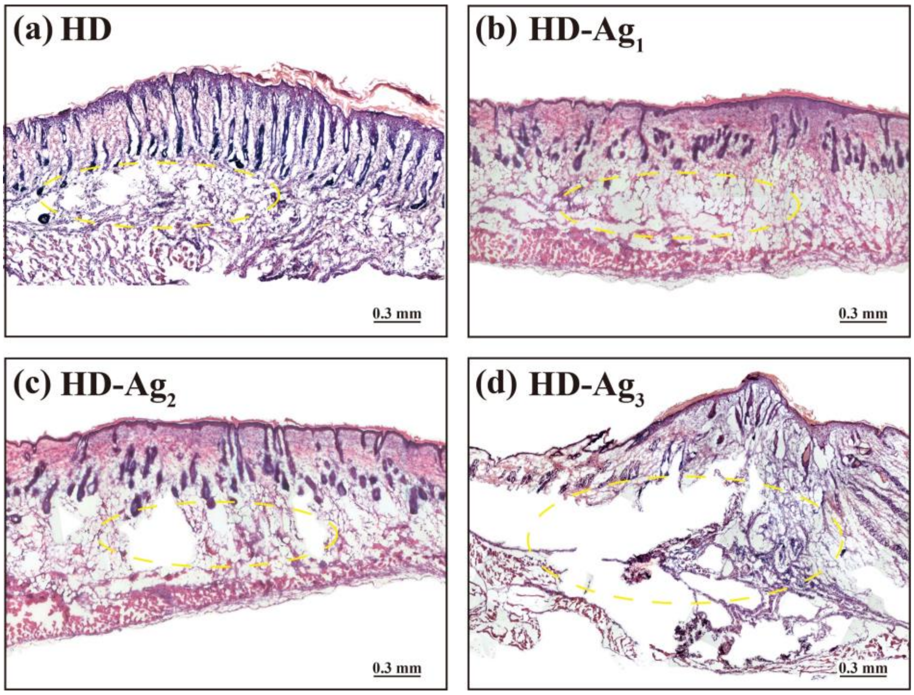

Figure S10. Histological analysis of the biocompatibility and degradation behavior of HD and HD-Ag hydrogels in vivo was performed after seven days of implantation. The implanted hydrogel sample was indicated by a yellow dotted cycle to denote its location.

Antibacterial Properties of HD-Ag Hydrogel

To assess the antibacterial efficacy of HD-Ag hydrogels, a zone of inhibition (ZOI) assay was conducted on both MRSA and PAO1 bacteria. The results indicated that no visible inhibition zone formed around HD hydrogel while clear zones were observed surrounding the hydrogels containing AgNPs, the size enlarging as AgNPs content increasing (Figure 3e). These results suggest that the leaching AgNPs out from the hydrogel matrix is what enables such inhibitory properties against MRSA and PAO1 bacteria. The serial dilution spread plate assay provided quantitative statistical results which corroborated those obtained in the ZOI test (Figure 3f). After co-cultivation for 18 h, HD hydrogels showed minimal antibacterial effect (~3×10^8 CFU mL^{-1} bacteria remained); whereas an appreciable decrease was observed in the quantity of MRSA and PAO1 in the HD-Ag groups to 1/10,000 of that found in Blank and HD group. These findings demonstrate that HD-Ag hydrogels possess desirable antimicrobial properties, making them a suitable dressing material for diabetic wound.

In Vivo Effect of the PMH Dressing on Bacterial-infected Diabetic Wound Healing

The effectiveness of the photovoltaic microcurrent hydrogel (PMH) dressing on skin wounds infected with a polymicrobial population, including Methicillin-resistant *Staphylococcus aureus* (MRSA) and *Pseudomonas aeruginosa* (PAO), was assessed in a

diabetic mouse model. The model was established via a regimen of a high-fat diet for three weeks, subsequent intraperitoneal injections of STZ for five consecutive days, and the maintenance of elevated blood glucose levels for six weeks. As shown in **Figure S11**a, there was a marked increase in the mice's blood glucose levels after the STZ injection, and these high levels were maintained at 15-25 mM for six weeks to allow the manifestation of diabetes-induced pathophysiological effects.[32] During the high-fat diet period, the mice experienced an increase in body weight from approximately 21.87 ± 0.63 g to 33.23 ± 0.87 g. A decline in body weight was noted following the STZ injection and the maintenance of high blood glucose levels, stabilizing finally at about 28.24 ± 0.80 g, approximately 15% lower than the peak. This is considered ethically permissible as it represents less than 20% of their original body weight prior to the treatment (Figure S11b) [33]. The PMH dressing was attached to the wound site on the back of the diabetic mice, and the healing process was initiated upon exposure to light (Figure S11c).

Figure 4b shows representative wound transitions over time. Statistical analysis revealed that from day 7 onwards there was significantly accelerated reduction in average wound area in the PMH dressing group compared with other groups (p<0.05; Figure 4f). It is worth noting that wounds in the PMH dressing group exhibited a more rapid closure than those in both PM patch and HD-Ag_2 group, suggesting that the exogenous electrical stimulation from the dressing promoted wound repair. Histological analysis provides a more detailed perspective of alteration. As illustrated in Figure 4d, markedly elevated levels of neutrophils infiltrated into the Blank and PM patch groups, demonstrating that inflammation persisted within the wounded area. Nevertheless, inflammation appeared to be generally diminished, concurrently with fibroblasts being present in high quantities within both HD-Ag_2 and PMH dressing groups. In addition, epidermis had almost regenerated completely in the wounds treated with PMH dressing (the yellow dashed line indicates regenerated epithelium), yet it had partially formed in HD-Ag_2 group. Contrarily, there was limited epidermal regeneration observed in both the Blank and PM patch groups (Figure 4d). The results of statistical analysis revealed that the average re-epithelization percentage of the wounds treated with PMH dressing was ~86%, showing a significant improvement (p<0.05) compared with other groups (Figure 4g).

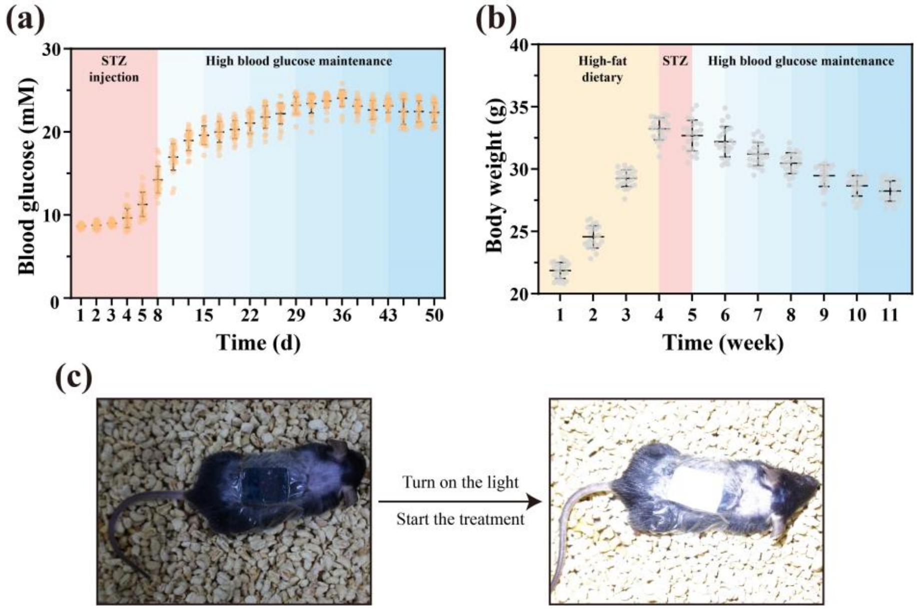

Figure S11. (a) The blood glucose monitoring results during the establishment of diabetic mouse animal models (starting from the first day of STZ injection). (b) The weight monitoring results during the establishment of the diabetic mouse animal model (starting from the first day of high-fat diet). (c) Representative images showing diabetic mice with infected wounds getting treated with PMH dressing.

Quantitative Label-free Global Proteomic Analysis

We have conducted label-free proteomics (LFP) to quantify the differentially expressed proteins (DEPs) between the samples of PMH dressing and the Blank group (treated with Tegaderm™ film dressing). LFP enables high throughput analyses of multiple proteins from a single sample or multiple samples. This technology holds immense potential for elucidating various molecular cues that are linked to tissue repair and can offer an accurate depiction of how proteins play a role in this complex process. The protein abundance heatmap for all proteins detected in wound tissues was provided, with a total of 4066 proteins identified (Figure S11d). The wound treated with PMH dressing generated a perturbed protein response consisting of 2567 DEPs ($p<0.05$); 2280 were upregulated (based on a cutoff value of 2-fold) and 287 were downregulated (based on a cutoff value of 0.5-fold), as represented by the volcano map in (Figure S11e).

Immunofluorescence Staining and mRNA Expression of Macrophage Polarization Markers

We used immunofluorescence experiments to evaluate macrophage polarization marker proteins (including M1 polarization marker iNOS[34] and M2 polarization marker TGF-β_1[35]) in the tissues at the mid-stage (15 days) of chronic wound healing. The results revealed

significantly higher expression of iNOS in the Blank and PM patch groups compared with the HD-Ag and PMH dressing groups (**Figure S12**a,c). Simultaneously, the HD-Ag and PMH dressing groups exhibited higher levels of TGF-β_1 than the Blank and PM patch groups (Figure S12b,f). These results suggest a certain promotive effect on M2 polarization of macrophages in chronic wounds treated with HD-Ag hydrogel. Further evidence supporting this conclusion was provided by the analysis of mRNA expression in wound tissues. In the Blank and PM patch groups, the mRNA expression of pro-inflammatory factors (TNF-α and IL-1β) was significantly higher than those in the HD-Ag_2 and PMH dressing groups during both the early (7d) and mid-stages (15d) of healing (Figure S12d-e). In contrast, the mRNA expression of anti-inflammatory mediators (TGF-β_1 and IL-10) was reversed (Figure S12g-h). These experimental results demonstrate that HD-Ag_2 has an immunomodulatory effect on diabetic wounds with persistent inflammation. It can promote the polarization of macrophages towards the M2 type and enhance the secretion of anti-inflammatory factors, accelerating the transition of the wound from an inflammatory state to a repair phase.

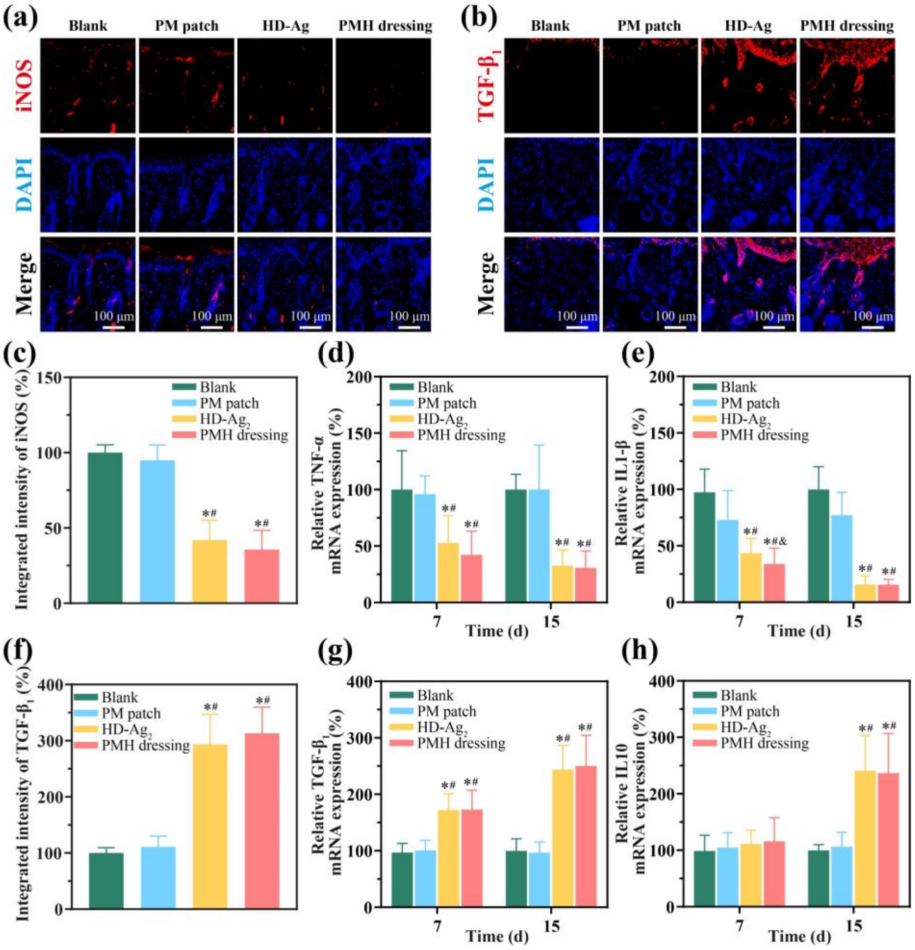

Figure S12. (a-b) The results of the immunofluorescence staining of iNOS and TGF-β_1 in skin wound tissues during the middle stage of healing (Day 15). (c) Quantitative analysis of iNOS fluorescence intensity in the tissues, compared with the blank group (normalized to 100%). (d-e) The relative mRNA expression of TNF-α and IL-1β in tissues during the early (7 days) and mid (15 days) stages of healing, compared with the blank group (normalized to 100%). (f) Quantitative analysis of the fluorescence intensity of TGF-β_1 in the tissues compared with the blank group (normalized to 100%). (g-h) The relative mRNA expression of TGF-β_1 and IL-10 in tissues during the early (7 days) and mid (15 days) stages of healing, compared with the blank group (normalized to 100%). Data were shown as mean ± SD (n=3, * p<0.05 when compared with Blank group; # p<0.05 when compared with PM patch group; & p<0.05 when compared with HD-Ag_2 group).

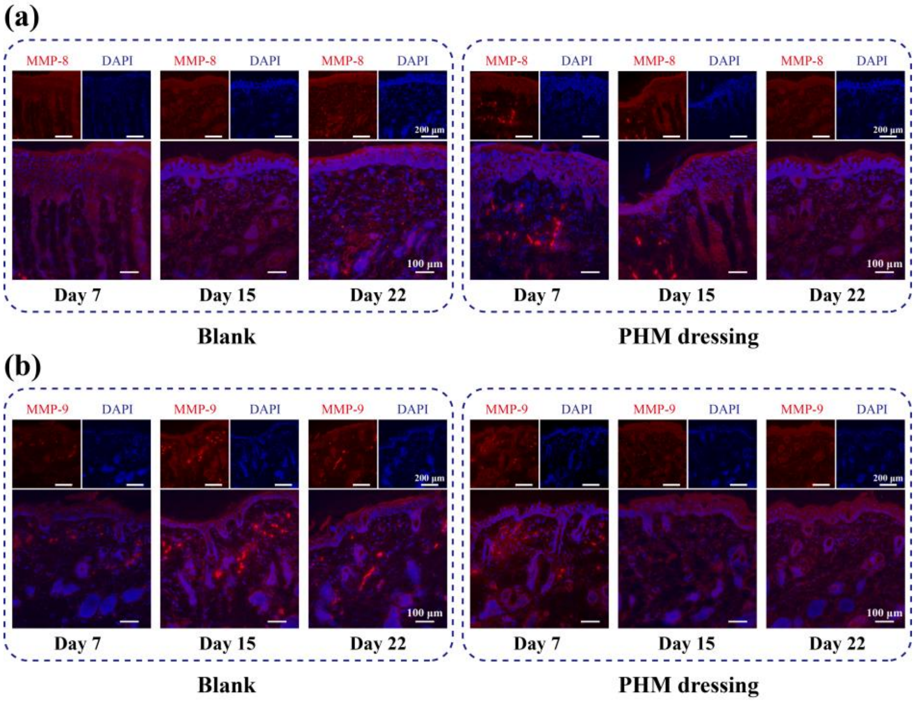

Figure S13. Immunofluorescence analysis revealing differential expression patterns of two matrix metalloproteinases (MMPs) during distinct phases of wound healing. (a) MMP-8, (b) MMP-9.

Table S4. Details of the identification of proteins with high differential expression.

No.	Accession Number	Protein Name	Gene	*p*-value	Fold Change	Main Function
1	Q99MQ4	Asporin	*Aspn*	$1.37E^{-05}$	2.35	Critical regulator of TGF-beta
2	P28653	Biglycan	*Bgn*	$4.47E^{-06}$	2.93	Involved in collagen fiber assembly
3	P14211	Calreticulin	*Calr*	$4.07E^{-06}$	2.96	Involved in protein folding and oligomeric assembly
4	P11087	Collagen type I, alpha 1	*Col1a1*	$5.60E^{-06}$	2.58	Structural constituent of extracellular matrix
5	Q01149	Collagen type I, alpha 2	*Col1a2*	$3.48E^{-06}$	4.24	Structural constituent of extracellular matrix
6	P08121	Collagen type III, alpha 1	*Col3a1*	$3.19E^{-06}$	2.99	Structural constituent of extracellular matrix
7	P02463	Collagen type IV, alpha 1	*Col4a1*	$5.04E^{-03}$	2.99	Structural constituent of extracellular matrix
8	P08122	Collagen type IV, alpha 2	*Col4a2*	$6.56E^{-06}$	4.21	Structural constituent of extracellular matrix
9	Q04857	Collagen type VI, alpha 1	*Col6a1*	$8.69E^{-06}$	2.57	Structural constituent of extracellular matrix
10	Q02788	Collagen type VI, alpha 2	*Col6a2*	$4.46E^{-06}$	2.68	Structural constituent of extracellular matrix
11	Q8C6K9	Collagen type VI, alpha 6	*Col6a6*	$1.50E^{-05}$	2.75	Structural constituent of extracellular matrix
12	Q80X19	Collagen type XIV, alpha 1	*Col14a1*	$1.98E^{-06}$	3.47	Structural constituent of extracellular matrix
13	Q9D1D6	Collagen triple helix repeat containing 1	*Cthrc1*	$9.00E^{-06}$	2.69	Regulator of collagen matrix deposition
14	P28654	Decorin	*Dcn*	$2.95E^{-06}$	4.62	Involved in fibrillogenesis
15	Q9QZZ6	Dermatopontin	*Dpt*	$2.26E^{-05}$	5.15	Accelerates collagen fibril formation, component of the intercellular junction
16	P54320	Elastin	*Eln*	$3.50E^{-06}$	4.71	Structural protein of tissues
17	Q61554	Fibrillin-1	*Fbn1*	$2.48E^{-06}$	4.38	Structural component of calcium-binding microfibrils
18	Q60675	Laminin subunit alpha-2	*Lama2*	$8.74E^{-06}$	11.85	Interacting with extracellular matrix to mediate the attachment, migration and organization of cells
19	P02469	Laminin subunit beta-1	*Lamb1*	$7.38E^{-06}$	2.42	Interacting with extracellular matrix to mediate the attachment, migration and organization of cells
20	P02468	Laminin subunit gamma-1	*Lamc1*	$1.03E^{-04}$	5.25	Interacting with extracellular matrix to mediate the attachment, migration and organization of cells
21	P16045	Galectin-1	*Lgals1*	$1.79E^{-06}$	4.04	Modulating cell-cell and cell-matrix interactions
22	Q07797	Galectin-3-binding protein	*Lgals3bp*	$7.86E^{-06}$	2.88	Promotes integrin-mediated cell adhesion
23	P11152	Lipoprotein lipase	*Lpl*	$7.25E^{-05}$	4.37	Enzyme in triglyceride metabolism
24	Q8K4G1	Latent-transforming growth factor beta-binding protein 4	*Ltbp4*	$3.39E^{-05}$	10.44	Regulator of TGF-beta activation
25	P51885	Lumican	*Lum*	$2.10E^{-06}$	3.99	A keratan sulfate proteoglycan involved in collagen fibril organization
26	P55002	Microfibrillar-associated protein 2	*Mfap2*	$3.69E^{-04}$	2.90	Component of the elastin-associated microfibrils

Table S4 (continued). Details of the identification of proteins with high differential expression.

No.	Accession Number	Protein Name	Gene	*p*-value	Fold Change	Main Function
27	Q9D1H9	Microfibril-associated glycoprotein 4	*Mfap4*	$2.17E^{-04}$	3.05	Contribute to the elastic fiber assembly and/or maintenance
28	O88322	Nidogen-2	*Nid2*	$6.56E^{-06}$	3.21	A basal lamina protein
29	Q62000	Mimecan	*Ogn*	$4.14E^{-04}$	3.38	Conjunction with TGF-beta
30	Q3V1T4	Prolyl 3-hydroxylase 1	*P3h1*	$1.10E^{-04}$	2.87	Basement membrane-associated chondroitin sulfate proteoglycan
31	Q7TQ62	Podocan	*Podn*	$5.27E^{-05}$	3.02	Regulator of cell proliferation and migration
32	Q9JK53	Prolargin	*Prelp*	$9.59E^{-07}$	13.26	Anchoring agents that connect the basement membrane to the connective tissue
33	P97347	Repetin	*Rptn*	$7.85E^{-06}$	2.91	A multifunctional epidermal matrix protein functioning in the cornified cell envelope formation
34	P07214	Secreted acidic cysteine rich glycoprotein	*Sparc*	$6.21E^{-06}$	3.03	Regulate cell growth through interactions with the extracellular matrix
35	Q8VCC9	Spondin 1	*Spon1*	$2.55E^{-03}$	3.22	Cell adhesion protein
36	P21981	Tissue transglutaminase	*Tgm2*	$6.59E^{-06}$	4.69	An enzyme that catalyzes cross-linking between proteins in the extracellular matrix, enabling scaffold structure formation

Table S5. Details of the identification of proteins with low differential expression.

No.	Accession Number	Protein Name	Gene	*p*-value	Fold Change	Main Function
1	Q07456	Alpha 1 microglobulin/bikunin precursor	*Ambp*	$3.74E^{-06}$	0.32	Heme-binding plasma glycoprotein
2	P11276	Fibronectin 1	*Fn1*	$1.55E^{-06}$	0.24	Deposited at the site of injury, forming a blood clot that stops bleeding
3	Q61001	Laminin subunit alpha-5	*Lama5*	$7.66E^{-05}$	0.29	Interacting with extracellular matrix components
4	P28862	Matrix metallopeptidase 3	*Mmp3*	$2.30E^{-04}$	0.36	Involved in the breakdown of extracellular matrix proteins
5	O70138	Matrix metallopeptidase 8	*Mmp8*	$1.97E^{-06}$	0.17	Involved in the breakdown of extracellular matrix proteins
6	P41245	Matrix metallopeptidase 9	*Mmp9*	$3.66E^{-06}$	0.20	Involved in the breakdown of extracellular matrix proteins
7	P35441	Thrombospondin 1	*Thbs1*	$1.93E^{-06}$	0.27	Platelet aggregation and angiogenesis
8	Q8R2Z5	Von Willebrand factor A domain-related protein	*Vwa1*	$1.90E^{-06}$	0.13	A protein that is part of the von Willebrand factor A domain superfamily
9	Q8CIZ8	Von Willebrand factor	*Vwf*	$1.83E^{-03}$	0.33	Promotes adhesion of platelets to the sites of vascular injury

References

[1] S. G. Gurgen, O. Sayin, F. Cetin, A. Tuc Yucel, *Inflammation* **2014**, *37*, 775.
[2] Z. Khalil, M. Merhi, *J Gerontol A Biol Sci Med Sci* **2000**, *55*, B257.
[3] Y. Liang, H. Tian, J. Liu, Y. Lv, Y. Wang, J. Zhang, Y. Huang, *Bioelectrochemistry* **2020**, *135*, 107578.
[4] Y. Chen, Y. Liang, J. Liu, J. Yang, N. Jia, C. Zhu, J. Zhang, *Biomater Sci* **2021**, *9*, 238.
[5] L. Wang, L. Mao, F. Qi, X. Li, M. W. Ullah, M. Zhao, Z. Shi, G. Yang, *Chemical Engineering Journal* **2021**, *424*, 130563.
[6] L. Mao, S. Hu, Y. Gao, L. Wang, W. Zhao, L. Fu, H. Cheng, L. Xia, S. Xie, W. Ye, Z. Shi, G. Yang, *Adv Healthc Mater* **2020**, *9*, e2000872.
[7] H. K. Jang, J. Y. Oh, G. J. Jeong, T. J. Lee, G. B. Im, J. R. Lee, J. K. Yoon, D. I. Kim, B. S. Kim, S. H. Bhang, T. I. Lee, *Int J Mol Sci* **2018**, *19*, 3025.
[8] J. Min, S. Demchyshyn, J. R. Sempionatto, Y. Song, B. Hailegnaw, C. Xu, Y. Yang, S. Solomon, C. Putz, L. E. Lehner, *Nature Electronics* **2023**, *6*, 630.
[9] M. Jakešová, T. A. Sjöström, V. Đerek, D. Poxson, M. Berggren, E. D. Głowacki, D. T. Simon, *npj Flexible Electronics* **2019**, *3*, 14.
[10] J. F. Maya-Vetencourt, G. Manfredi, M. Mete, E. Colombo, M. Bramini, S. Di Marco, D. Shmal, G. Mantero, M. Dipalo, A. Rocchi, M. L. DiFrancesco, E. D. Papaleo, A. Russo, J. Barsotti, C. Eleftheriou, F. Di Maria, V. Cossu, F. Piazza, L. Emionite, F. Ticconi, C. Marini, G. Sambuceti, G. Pertile, G. Lanzani, F. Benfenati, *Nat Nanotechnol* **2020**, *15*, 698.
[11] Z. Li, H. Feng, Q. Zheng, H. Li, C. Zhao, H. Ouyang, S. Noreen, M. Yu, F. Su, R. Liu, *Nano Energy* **2018**, *54*, 390.
[12] W. Hu, X. Wei, L. Zhu, D. Yin, A. Wei, X. Bi, T. Liu, G. Zhou, Y. Qiang, X. Sun, *Nano Energy* **2019**, *57*, 600.
[13] R. Luo, Y. Liang, J. Yang, H. Feng, Y. Chen, X. Jiang, Z. Zhang, J. Liu, Y. Bai, J. Xue, S. Chao, Y. Xi, X. Liu, E. Wang, D. Luo, Z. Li, J. Zhang, *Adv Mater* **2023**, *35*, e2208395.
[14] C. Wang, Y. Hu, Y. Liu, Y. Shan, X. Qu, J. Xue, T. He, S. Cheng, H. Zhou, W. Liu, *Adv Funct Mater* **2023**, *33*, 2303696.
[15] S. Du, N. Zhou, Y. Gao, G. Xie, H. Du, H. Jiang, L. Zhang, J. Tao, J. Zhu, *Nano Research* **2020**, *13*, 2525.
[16] T. Zhang, H. Liang, Z. Wang, C. Qiu, Y. B. Peng, X. Zhu, J. Li, X. Ge, J. Xu, X. Huang, J. Tong, J. Ou-Yang, X. Yang, F. Li, B. Zhu, *Sci Adv* **2022**, *8*, eabk0159.
[17] P. Chen, C. Xu, P. Wu, K. Liu, F. Chen, Y. Chen, H. Dai, Z. Luo, *ACS Nano* **2022**, *16*, 16513.
[18] X. F. Wang, M. L. Li, Q. Q. Fang, W. Y. Zhao, D. Lou, Y. Y. Hu, J. Chen, X. Z. Wang, W. Q. Tan, *Bioact Mater* **2021**, *6*, 230.
[19] H. Guo, D. D'Andrea, J. Zhao, Y. Xu, Z. Qiao, L. E. Janes, N. K. Murthy, R. Li, Z. Xie, Z. Song, R. Meda, J. Koo, W. Bai, Y. S. Choi, S. W. Jordan, Y. Huang, C. K. Franz, J. A. Rogers, *Adv Funct Mater* **2021**, *31*, 2102724.
[20] Y. Huang, H. Li, T. Hu, J. Li, C. K. Yiu, J. Zhou, J. Li, X. Huang, K. Yao, X. Qiu, Y. Zhou, D. Li, B. Zhang, R. Shi, Y. Liu, T. H. Wong, M. Wu, H. Jia, Z. Gao, Z. Zhang, J. He, M. Zheng, E. Song, L. Wang, C. Xu, X. Yu, *Nano Lett* **2022**, *22*, 5944.
[21] a) G. Eke, N. Mangir, N. Hasirci, S. MacNeil, V. Hasirci, *Biomaterials* **2017**, *129*, 188; b) C. C. L. Schuurmans, M. Mihajlovic, C. Hiemstra, K. Ito, W. E. Hennink, T. Vermonden, *Biomaterials* **2021**, *268*, 120602.
[22] E. Hachet, H. Van Den Berghe, E. Bayma, M. R. Block, R. Auzely-Velty, *Biomacromolecules* **2012**, *13*, 1818.
[23] H. Lee, S. M. Dellatore, W. M. Miller, P. B. Messersmith, *Science* **2007**, *318*, 426.
[24] M. Rycenga, C. M. Cobley, J. Zeng, W. Li, C. H. Moran, Q. Zhang, D. Qin, Y. Xia, *Chem Rev* **2011**, *111*, 3669.

[25] A. GhavamiNejad, C. H. Park, C. S. Kim, *Biomacromolecules* **2016**, *17*, 1213.
[26] M. Rehan, A. Barhoum, G. Van Assche, A. Dufresne, L. Gatjen, R. Wilken, *Int J Biol Macromol* **2017**, *98*, 877.
[27] A. R. Gliga, S. Skoglund, I. O. Wallinder, B. Fadeel, H. L. Karlsson, *Part Fibre Toxicol* **2014**, *11*, 11.
[28] K. Sapkota, K. B. Narayanan, S. S. Han, *Journal of Cluster Science* **2017**, *28*, 1605.
[29] M. A. Raza, Z. Kanwal, A. Rauf, A. N. Sabri, S. Riaz, S. Naseem, *Nanomaterials* **2016**, *6*, 74.
[30] I. L. Bergin, L. A. Wilding, M. Morishita, K. Walacavage, A. P. Ault, J. L. Axson, D. I. Stark, S. A. Hashway, S. S. Capracotta, P. R. Leroueil, A. D. Maynard, M. A. Philbert, *Nanotoxicology* **2016**, *10*, 352.
[31] a) Y. Liang, B. Chen, M. Li, J. He, Z. Yin, B. Guo, *Biomacromolecules* **2020**, *21*, 1841; b) Y. Liu, Q. Ma, M. Yang, X. Dong, Y. Yang, J. Wang, W. Yu, G. Liu, *Chemical Engineering Journal* **2016**, *284*, 831.
[32] A. P. Sawaya, R. C. Stone, S. R. Brooks, I. Pastar, I. Jozic, K. Hasneen, K. O'Neill, S. Mehdizadeh, C. R. Head, N. Strbo, M. I. Morasso, M. Tomic-Canic, *Nat Commun* **2020**, *11*, 4678.
[33] D. S. Liu, C. P. Duong, S. Haupt, K. G. Montgomery, C. M. House, W. J. Azar, H. B. Pearson, O. M. Fisher, M. Read, G. R. Guerra, Y. Haupt, C. Cullinane, K. G. Wiman, L. Abrahmsen, W. A. Phillips, N. J. Clemons, *Nat Commun* **2017**, *8*, 14844.
[34] D. Luo, Y. Guo, Y. Cheng, J. Zhao, Y. Wang, J. Rong, *Aging (Albany NY)* **2017**, *9*, 2069.
[35] M. Arabpour, A. Saghazadeh, N. Rezaei, *Int Immunopharmacol* **2021**, *97*, 107823.
